# A van der Waals force-based adhesion study of stem cells exposed to cold atmospheric plasma jets

**DOI:** 10.1038/s41598-022-16277-1

**Published:** 2022-07-15

**Authors:** Kobra Hajizadeh, Hassan Mehdian, Kamal Hajisharifi, Eric Robert

**Affiliations:** 1 Physics department, Faculty of Science, South Tehran Branch, Islamic Azad University, Tehran, Iran; 2 School of Physics, Institute for Research in Fundamental Science (IPM), P. O. Box 19395-5531, Tehran, Iran; 3 Department of Physics and Institute for Plasma Research, Kharazmi University, Tehran, 15614 Iran; 4 UMR 7344, CNRS/Université d’Orléans, GREMI, Orléans, France; 5 Research Center for Modeling and Optimization in Science and Engineering, South Tehran Branch, Islamic Azad University, Tehran, Iran

**Keywords:** Biophysics, Permeation and transport

## Abstract

Cold atmospheric plasma has established its effect on cell adhesion. Given the importance of cell adhesion in stem cells, the current study investigates the effect of plasma treatment on Human Bone Marrow Mesenchymal Stem Cells (HBMMSCs) adhesion by which the differentiation and fate of cells are determined. In this paper, adhesion modification is considered not only for cell- ECM (Extra cellular Matrix), but also between suspended cells, and enhanced adhesions were found in both circumstances. Regarding the previous works, the increase of the cell–ECM adhesion during the plasma therapy was mostly attributed to the enhancement of the production and activity of integrin proteins. Nevertheless, considering the importance of van der Waals forces at the cellular level, the effect of cold plasma on VDWFs and so its effect on adhesion is investigated in this work for the first time, to the best of our knowledge. For this purpose, employing the semi-empirical methods, the role of the plasma therapy on the VDWF between the cells has been studied at three levels; (a) plasma-induced dipole formation, (b) Hammaker coefficient modification of culture medium, and c) cell roughness modification. For suspended cell condition, we conclude and support that van der Waals forces (VDWFs) enhancement has a key role in cell adhesion processes. We believe that, the present work gives a new physical insight in studying the plasma therapy method at the cellular level.

## Introduction

**Figure 1:**
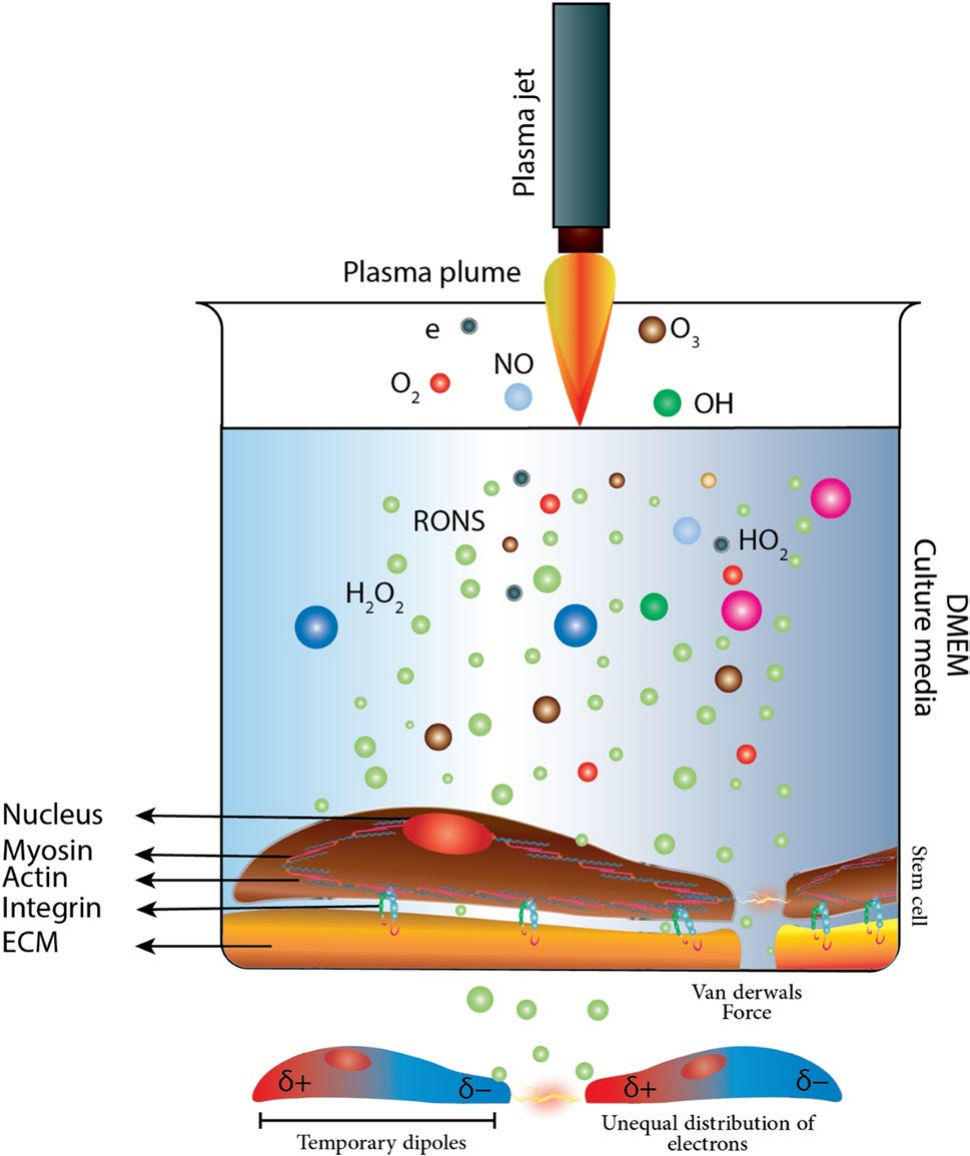
A schematic of Plasma interactions in aqueous media. Electric field, ions, electrons, neutrals, and reactive oxygen and nitrogen species is produced. The charge distribution of Cell is changed and creates electric dipoles.

Cold Atmospheric Plasma (CAP) with its great potential in various fields including medicine^1^, biology, and physiology^2,3^ has recently demonstrated its therapeutic effects, particularly in cancer therapy^4^ and wound healing^5–7^. Although the presence of reactive oxygen/nitrogen species (RONS) in the physical environment of the plasma plume and plasma-activated medium has been introduced as the primary factor in atmospheric plasma-based therapies, the effect of another crucial factor such as electric field inducing cell polarization leading to dipole formation is probably of key importance. In this regard, plasma can affect the van der Waals, VDW, forces between cell–cell and cell–ECM (Extra cellular Matrix) in two ways; (a) dipole generation, as a consequence of plasma-induced electric field and, (b) cell-surface roughness modification, caused by plasma treatment^8^. Moreover, the effect of RONS on cell–cell and cell–ECM adhesion illustrated schematically in Fig. 1 has been long-established by many authors since quite a long time^9–13^. Given the large amount of RONS produced during plasma therapy together with the formation of dipoles, it is very likely that media exposure to plasma affects cell adhesion and maybe that is the reason of rounded shape detachment pattern during the plasma therapy^14^.

The regulation of cell adhesion is of special importance for stem cells. Not only is cell differentiation influenced by cell shape at the end of the adhesion process, but also adhesion determines the timing of cell migration onset/cessation as well as the migration pathway^15^. On the other hand, cell adhesion is very essential in its communication and regulation^16^. The cell–ECM mechanical communications can manage and control cell performance and function^17,18^. The role of cell adhesion is much more crucial than merely binding two cell surfaces together or attaching cells to the ECM. Cell adhesion indeed triggers signaling pathways to activate and modulate cell function leading to physical changes in cell properties such as stiffness, motility, and cell–ECM interactions. There are various mechanisms of adhesion, including electrostatic interactions^15,19–22^. In this regard, Kendall and Robert^23^ showed that among the electrostatic forces, van der Waals is the key force involved in adhesion than any others. Despite the weakness of this force compared to hydrogen and ionic bonds, two important factors make it very effective in adhesion; (a) its ubiquity across all molecules and, (b) Cell size, for which the role of VDWF will be very significant^24,25^. Therefore, beside the established effect of RONS on cell–cell and cell–ECM adhesion^9–13^, any factor that affects the VDW force can be involved in cell adhesion, with the effect that, changes the behavior, function, and fate of cells^26,27^. The change of cell adhesion leads to a wide range of diseases, including osteoarthritis^28^, cancer^24,29,30^, osteoporosis^25,31^, and atherosclerosis (atherosclerosis)^32–34^. So, the investigation and control of cell adhesion should be accounted for, in plasma based therapies, given that any change of cell adhesion features might be considered as a warning indicator for cell health.

Stem cell adhesion is of two essential importance, firstly the selectivity of cell adhesion and secondly, its importance in biomaterials, which plays a crucial role in tissue and organ formation. Therefore, to develop any new therapeutic method (e.g. Plasma-based therapies); it is important to study its effects on adhesion of the cells. According to previous researches^34–36^, at a first glance, plasma can activate integrin, as one of the key molecules in cell adhesion. Haertel, in 2014 found that CAP affects integrin expression, so it can alter cell–ECM adhesion^32,33^.

The importance of focal adhesion (FA) originates from the fact that it is involved in actin cytoskeleton organization. FAs reveal physical cell–ECM contact as well as internal/external signaling^15^. Therefore, quantitative analysis of FAs is crucial for understanding important biological processes including cell shape, motility, cell proliferation, and differentiation^16,17^. Given that cells can form different FAs at the micrometer scale, their quantitative analysis requires optimized image analysis approaches.

The effect of plasma on cell adhesion could also result from polarization and consecutive dipoles formation (Fig. 1). Plasma can also contribute to cell adhesion modification through changing the VDWF between cell–cell and cell–ECM. Furthermore, plasma species may modify the surface roughness, which, in turn, affects VDWF [8]. To the best of our knowledge, the effect of plasma on VDWF between cells has not been studied yet, and so its likely effect on stem cell adhesion has not been fully elucidated. In this regard, in present paper, we try to address these two phenomena. De-adhesion, and shear stress assay, along with morphological studies, have been used to evaluate adhesion strength and cell elasticity. The conductivity of cell culture media is evaluated to study the VDWF modification.

## Materials and methods

### Cell culture

Human Bone Marrow Mesenchymal Stem Cells (HBMMSCs, provided from: Stem Cell Technology Research Center (STRC) Tehran, Iran, the flow cytometry data are provided in supplementary file S1) cultured at 37 $^\circ$C in a humidified atmosphere containing 5% CO$_2$ in DMEM-F12 (Sigma Aldrich) supplemented with 10% fetal bovine serum (FBS, Sigma Aldrich), and 1% penicillin/streptomycin (Sigma Aldrich). Cells were maintained in cell culture flasks 25 cm$^{2}$ (Bio-One) and the media was changed every three days. Having confirmed the cell confluence of 90% for cells in the flask, they were collected by trypsin—EDTA (Sigma Aldrich) and reseeded. Cells were transferred to 3.5 cm (in diameter)-Petri dishes after passage number 3, and some cells were cultured on coverslips for various objectives, as mentioned in the next sections. For different purposes that includes using trypsin (e.g. de-adhesion assay, Cell adhesion strength), prior to trypsinization, cells were cultured at 37 $^\circ$C    for 24 h to prepare them for assays. Some of the petri dishes were exposed directly to the plasma output of the CAP system, ignited at both high (35 W, representing a high risk area^37^) and low (20 W, representing a safe area^37^) power ranges of the generator for all purposes. The distance between plasma plume and the cell culture media was maintained to 5 mm, the gas flow rate to 1 slm, and the exposure time varied from 30 to 90 s in 10 s increments (i.e., 30, 40, ..., 90), just for one of the assays (cell adhesion strength) exposure time was extended to 120 s. On the other hand, using above-mentioned protocol, some cells were cultured on coverslips (coverslips were placed in 3.5 cm Petri dishes) to be treated with various doses of plasma-activated water, PAW, or exposed directly to plasma depending on the purposes that will be discussed in detail in the relevant sections. As previously stated^37^, the PAW has been classified as; (1) high dose: the power of plasma generator 35 W and the exposure time of 90 s and, (2) low dose: the power of plasma generator 20W with exposure time less than 90 s. After treatment, cells were incubated under the same conditions for another 24 h.

### Cold atmospheric plasma (CAP) device

The Argon-fed DBD (dielectric barrier discharge) plasma Jet of Kharazmi University-Plasma lab, which was utilized to treat Stem Cells, consisted of a central power electrode coupled to a radio frequency, 13.56 MHz-RF, through an impedance matching device. The core electrode, a 1 mm steel wire, is inserted within a 2 mm-thick quartz tube wrapped with Teflon tape and employed as a dielectric. The ground electrode is a steel electrode with a diameter of 5 mm. Plasma was produced using an input flow of around 1 slm of industrial argon gas (99.9999%)^37^. The power was set to 20 W and 35 W (as no-risk and high-risk limits of generator’s power for treating the stem cells^37^) and the gas flow rate was of 1 slm. The bulk plasma temperature was measured to be around 37 $^\circ$C. The optical emission spectrum of this Argon plasma plume can be found in reference^37^ which shows the generation of some important reactive species generated with this device (OH, O, NO). In all of the procedures, cells were treated with plasma while remaining in cell culture media (no matter if they were treated directly or with plasma-activated water, PAW). The distance between the plasma plume and the cell culture medium was fixed at 5 mm for direct exposure.

### DAPI staining and fluorescence imaging

Cells were stained with DAPI (4,6-diamidino-2-phenylindole) according to Cold Harbor Spring protocol to follow the cell dynamics and shapes.

DAPI, a DNA-staining blue fluorescent dye, can be activated and pass through both healthy and stable cell membranes, with a lower luminosity for live cells than the fixed membrane. DMEM was discarded from the petri dishes and the cells were washed in PBS (phosphate-buffered saline), according to [Cold Spring Harbor] protocol to prepare them for DAPI staining. Afterwards, cells were fixed in 4% paraformaldehyde for 10min, permeabilized with 0.5% Triton X-100 for 15min at room temperature, washed three times with PBS, and incubated for another 5 min. Thereafter, the cells were washed three times with PBS again, 50 $\upmu$l DAPI (1:500, Sigma-Aldrich) added to each well, and incubated for another 5 min. Finally, each well added 50 $\upmu$l of PBS to maintain the cells hydrated throughout imaging. A confocal microscope was used to monitor and record images of stained cells (Zeiss LSM 510 microscope). The samples were mounted on the stage of the microscope, using slides. Imaging was performed using a Nikon microscope equipped with an incubator to control temperature, humidity, and CO$_2$. Images were recorded with a CCD camera. The stained cells were used in Focal Adhesion (FA) assay and also in “Shear stress assay results” to find the duplet cells (N$_2$) and single cells (N$_1$).

### De-adhesion assay

The resistance of cells for detachment from the substrate, called de-adhesion, is used as a suitable parameter to measure cell adhesion. Mechanical forces that are able to separate cells from the substrate are used as a tool to determine the extent of cell adhesion^38,39^. In this study, to quantify cell adhesion dynamics, use has been made of“de-adhesion assay”^40^, in which the cells were separated from the external matrix with trypsin and cellular contraction was observed before whole-cell detachment. To quantify the kinetics of cell contraction, the average normalized area of cell contact with the substrate is evaluated temporally. In this regard, the surface area of the cells was calculated in a time-dependent manner. The difference between the cell area in time t, A$_\text{ t }$, and the initial one, A$_\text {initial}$, (i.e., A$_\text {initial}$ - A$_\text{ t }$) normalized to the difference between the initial and final cell surface, A$_\text {final}$, (i.e., A$_\text {initial}$ - A$_\text {final}$) were measured at each time. Finally, the time constants were calculated by fitting the normalized cell surface area vs. time into a sigmoid curve.

**Figure 2:**
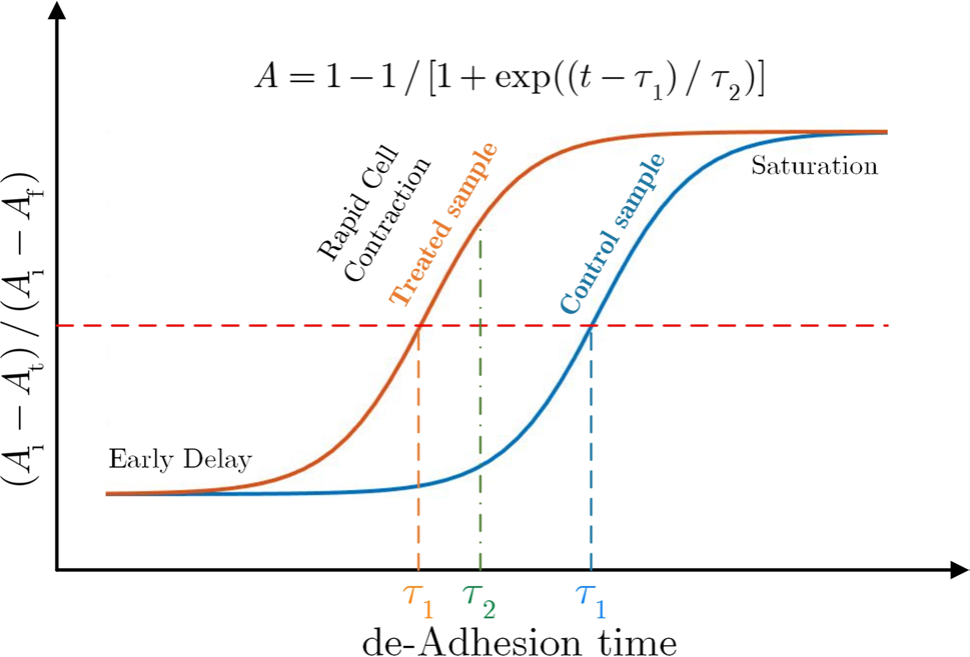
The normalized cell surface area versus de-adhesion time for treated and untreated (control) cells.

An example can be found in Fig. 2, where treated (red curve) and untreated (blue curve) cells make sigmoid curves and each of them consists of three distinct stages: early delay, rapid cell contraction, and saturation. Two time-constants apparent in this diagram $\tau _1$ and $\tau _2$, indicate cell adhesion and cell elasticity, respectively. The first time constant, $\tau _1$, is the time to decrease the cell surface area to half, and the second time constant, $\tau _2$, demonstrates the reduction of another 25% of the cell surface area to reach 75% of the total area.

### Focal adhesion

To compare Focal Adhesion (FA) formation before and after plasma treatment, stem cells were prepared using same protocol as described in “Cell culture”. In this regard, after passage no: 3, the cells seeded on coverslips (while the coverslips are placed in petri-dishes), incubated at 37 $^\circ$C    in a humidified atmosphere containing 5% CO$_2$ in DMEM-F12 (Sigma Aldrich) for 24 h. Hereafter, the cell media exposed to plasma directly at various time duration (30, 50, 70, 90, 100, and 120 s), while the distance between plasma plume and cell media was kept at 5 mm, as mentioned in “Cell culture”. The power of plasma generator was set at 20 W. The cells were again incubated at 37 $^\circ$C    for 24 h. The Control groups were divided into two groups of four. The first group was stained with DAPI (according to Sigma-Aldrich cell staining protocols, mentioned in “DAPI staining and fluorescence imaging”) as soon as the cells adhered to the plate, but before FA formation. The other group was treated with Argon while the plasma was off. The cells were all stained with DAPI, using same protocol as “DAPI staining and fluorescence imaging”. Use has been made of a CCD camera coupled to Nikon L150 Microscope, their images were recorded. Counting the focal points became possible after converting the images to an 8-bit format and subtracting background by combining CLAHE and LOG-3D filter plugins. Analysis stages were carried out by integrating LOG3D and CLAHE plugins from the NIH (National Institutes of Health) program^41^. The steps required to enable quantitative measurement of FA can be found in supplementary file S1.

### De-adhesion dynamics of cells

**Figure 3:**
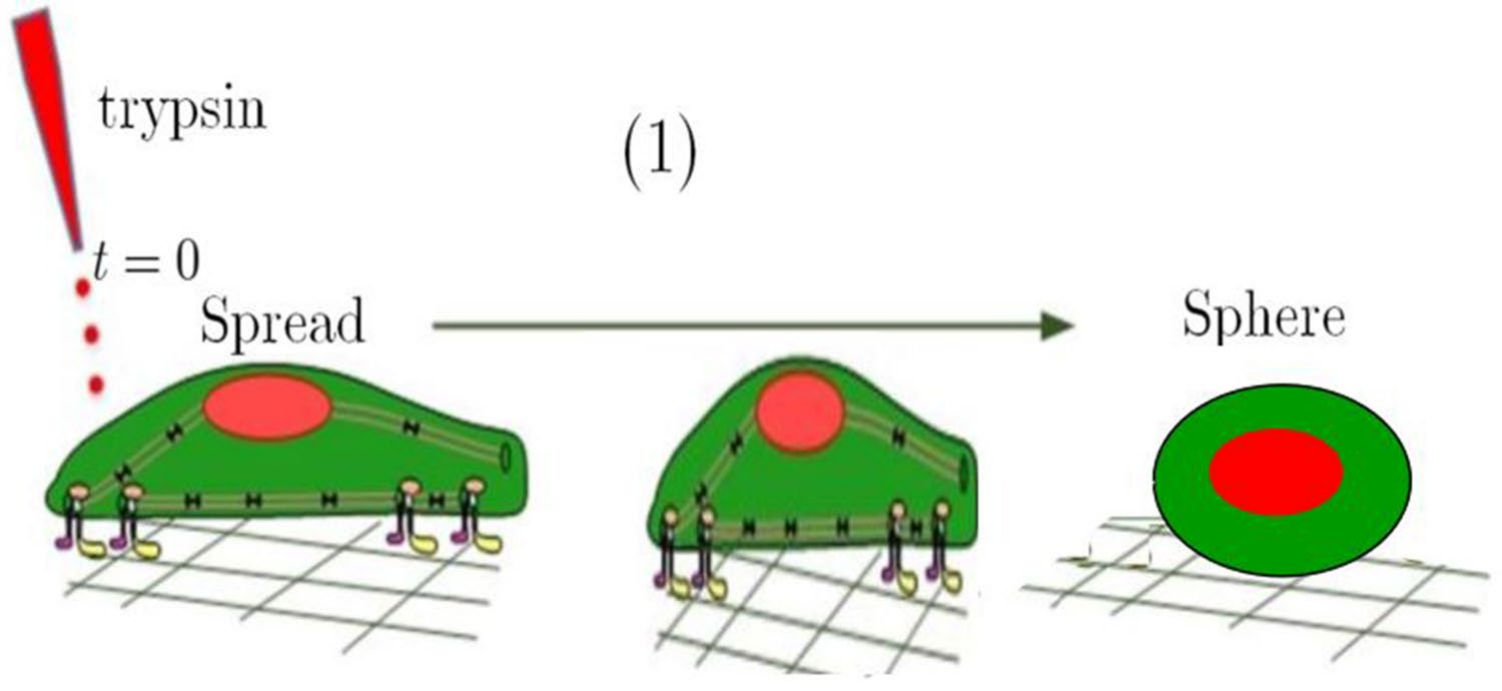
Schematic of de-adhesion assay.

De-adhesion experiments were performed, according to Sen^40^. To this end, cell resistance to de-adhesion was evaluated, and the adhesion strength coefficient was determined. A schematic of de-adhesion assay can be seen in Fig. 3. In summary, cells were prepared as “Cell culture”, then the cells were seeded on coverslips, incubated for 24 h, and then treated with plasma directly, as described previously. At the next step, treated cells were washed with 1 $\times$ PBS; afterward, coverslips were mounted on the stage of a microscope. At the last step, they were incubated with 0.25% trypsin-EDTA.

The cell detachment assay had two steps: First, Trypsin-EDTA utilized to detach focal adhesion contacts between cells and ECM. Next, cell contraction was observed until rounding up and separating from the substrate. In the meanwhile, images of both treated and non-treated cells were recorded every 15 s and analyzed with a canon microscope. The surface area of the cells was calculated in a time-dependent manner. The difference between the cell area in time t and the initial one (i.e., A$_\text {initial}$ - A$_\text {t}$) normalized to the difference between the initial and final cell surface (i.e., A$_\text {initial}$ - A$_\text {final}$) were measured at each time. The images were recorded at a 15 s interval at 40 $\times$ magnification until the cells were collected (rounded) but still remained attached to the substrate (with no further apparent change in the spread area). Images were processed in NIH (National Institutes of Health-Laboratory). To determine the effect of plasma treatment on the de-adhesion time, at least 30 cells were examined in three independent experiments and repeated for both high- and low-dose conditions. Student’s t-test and one-way ANOVA have been employed to determine the statistical validity of the results. t P-and P-values for each set of results are listed in the corresponding captions.

### Shear stress assay

A microfluidic channel was created using soft lithography:

**(A)** A spin-coating with a negative light-resistance was applied to the silicon wafer, which was then soft baked for a few minutes. It was placed under a mask and put on a hot plate for a few minutes after exposure, preceded by a short resting time, using a wafer aligner. Following that, the wafer was expanded at ambient temperature prior to getting washed with isopropyl alcohol (IPA) to eliminate any leftover wafer residue.

**(B)** The mold is filled with a 10:1 (weight) mixture of Polydimethylsiloxane, PDMS (liquid) and cross-linking agent (to cure the PDMS) and heated to a high temperature (70 $^\circ$C    for 1 h). The PDMS can be removed from the mold once it has solidified. In the PDMS block, a micro-channel has now been created.

**(C)** The cooked PDMS channel was separated from the original, cut, and punched to join the micro-tube. PDMS devices were directly bonded to a glass substrate without any surface preparation and then treated with oxygen plasma. To enable fluid injection, the microfluidic device’s inputs and outputs are punched with a PDMS puncher. Finally, Oxygen plasma (using an RF generator, at the power of 80W, for 1 min) is used to treat the glass slide and the face of the PDMS block with micro-channels. The microfluidic chip can now be sealed with PDMS and glass bonding thanks to the plasma treatment.

**Figure 4:**
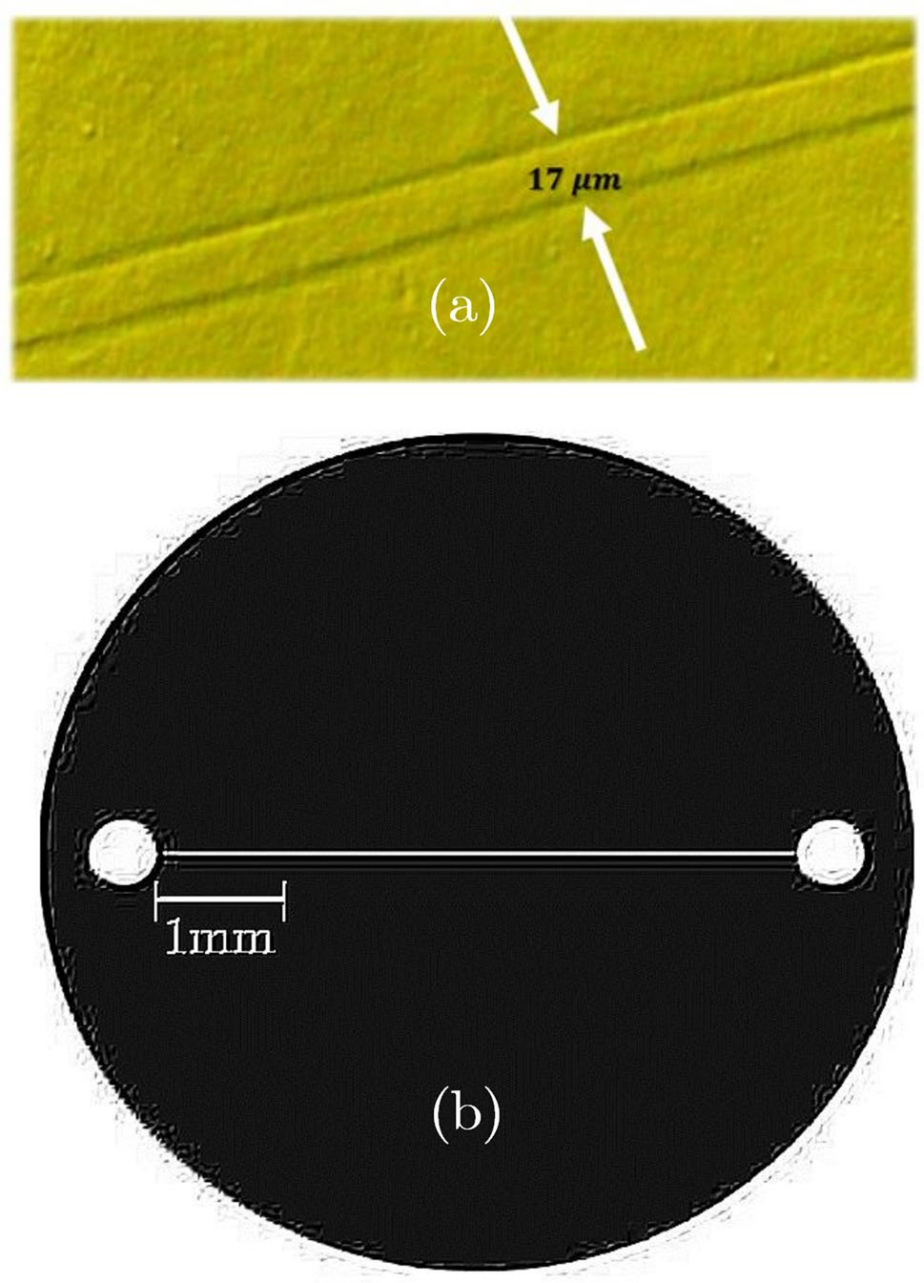
(**a**) Shear stress channel embedded in the polydimethylsiloxane, PDMS, (**b**) embedded 17-micron channel to create shear stress.

Figure 4a,b show the micro-scale channel embedded in PDMS polydimethylsiloxane to generate the flow. Dimensions of PDMS were 1cm in length, 1cm in width, and 0.5cm in height.

**Figure 5:**
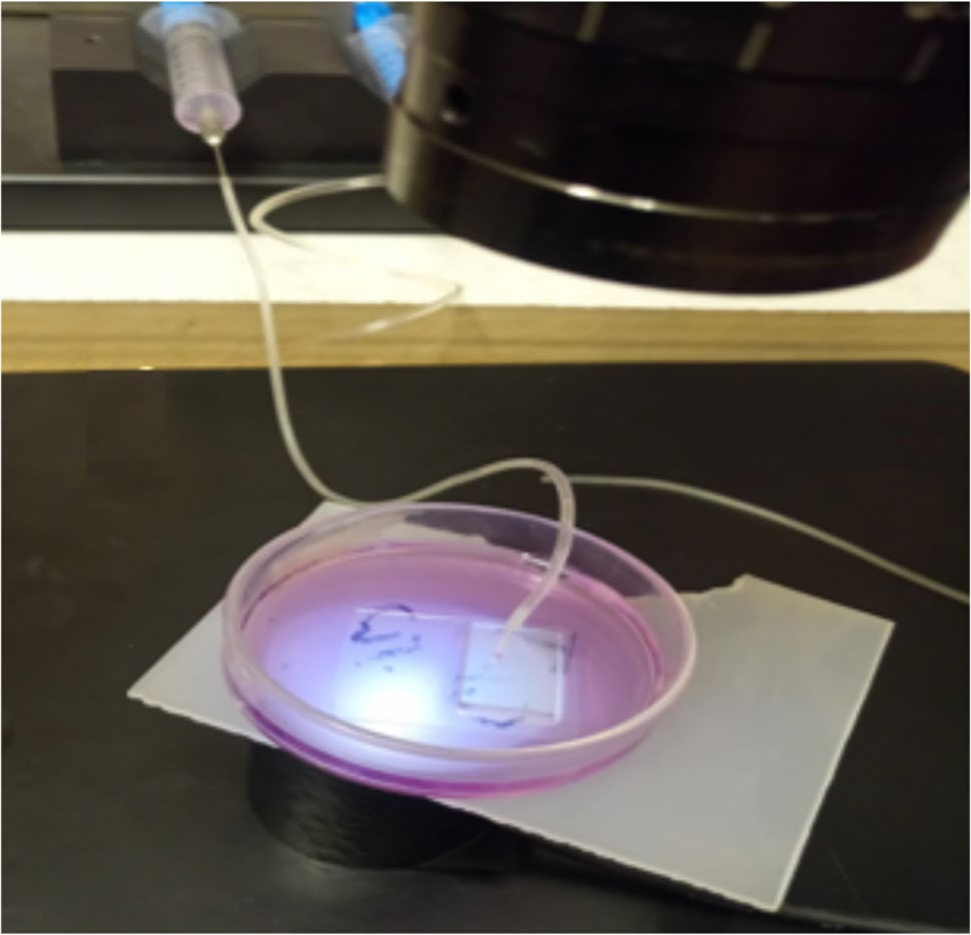
Connecting the syringe pump to the micro-channel and making stress via the 17-micron channel in PDMS.

**(D)** Shear stress assay: The shear stress assay is used to investigate the effects of shear flows on cells. A syringe pump would provide the shear flow, which would then reach the cells through the micro-channel. Cell culture media is used to apply shear stress on cells, which increases progressively until half of the cells separate from the substrate. Cells were cultured and passaged as described in “Cell culture”. The cells were seeded on a petri-dish in which the slide and mounted micro-channel (made into PDMS) were inserted prior to cell sediment, after passage 3. In fact, the cells were cultured on a slide to which the micro-channel was attached, causing shear stress to be applied directly to the cells by the flow passing through the micro-channel. The aim is to study the flow impact of the syringe pump, which is applied to the cell through the micro-channel. To this end, after seeding the cells, they were incubated for two days at 37 $^\circ$C    under the conditions described in “Cell culture”, to ensure that the cells adhered to the substrate. Using Teflon tubing, the outlet of the channel was connected to the syringe pump. A proper flow was produced by the pump in the micro-channel by filling the micro-channel (17 microns in diameter) inlet with cell culture media and pumping at a high flow rate (40 $\upmu$L/min) for 3 min, as documented in Fig. 5. In this paper, the syringe pump produces shear stress ranging from 15 to 45 dyne per square meter (the cell culture media were flushed by the syringe pump). The device was put on the stage of an upright microscope (Nikon L150) and the channel region was made visible with its“40 $\times$”objective for shooting photographs using a CCD camera connected to the microscope. Pictures were taken before and after the experiment. Use has been made of Imagej cell counter plugin (by clicking the cell image, the cell counter plugin allows to count cells manually) to count attached cells. The adhered cells were counted to determine the residual proportion. Finally, the fraction of adhered cells was shown as a function of shear stress intensity. The physical adhesion strength of each cell was determined using the critical shear stress, at which only 50% of the cells remained.

### Cell–cell adhesion strength

All methodologies detailed in the earlier sections of this paper have been focused on the cell–ECM adhesion and its associated assays (say, de-adhesion assay, FA assay). Given that the reported enhanced adhesion might have been attributed to the higher integrin proteins activity and expression, according to Keidar, and Weltmann^42^, as well as considering that the focus of this paper is primarily on cell–cell adhesion, seemingly, it is preferable to eliminate the effect of substrate-adhesion by removing the substrate and treating the cells as the cell suspension, instead. Consequently, we employed the Kendall approach^23^ in treating the cell suspension rather than cell on substrate. To this end, trypsin was used to detach the cells from the substrate and collect them on vials, following which trypsin was inactivated by Trypsin Neutralization Solution (TNS), then cell culture media was added to vials, and finally the cells were exposed to plasma directly (at different exposure times) while immersed in cell culture media. According to Kendall^23^, if the cell–cell adhesion is increased, (by raising van der Waals force between them), the cells will become more adherent to one another and form couples, increasing the proportion of double-cells to singlets. The strength of cell–cell adhesion increases with the couple to single ratio. Therefore, in this investigation, the number of coupled and single cells are required which which was proved challenging for immersed cells. As a result, to facilitate cell counting, we decided to mount cells on a slide and use a fluorescent dye like DAPI to stain them.

Subsequently, to make counting coupled and single cells possible, the cells were placed on a slide, stained with DAPI according to the protocol in “Focal adhesion”, and photographed using a CCD camera attached to a Nikon fluorescence microscope.

**Figure 6:**
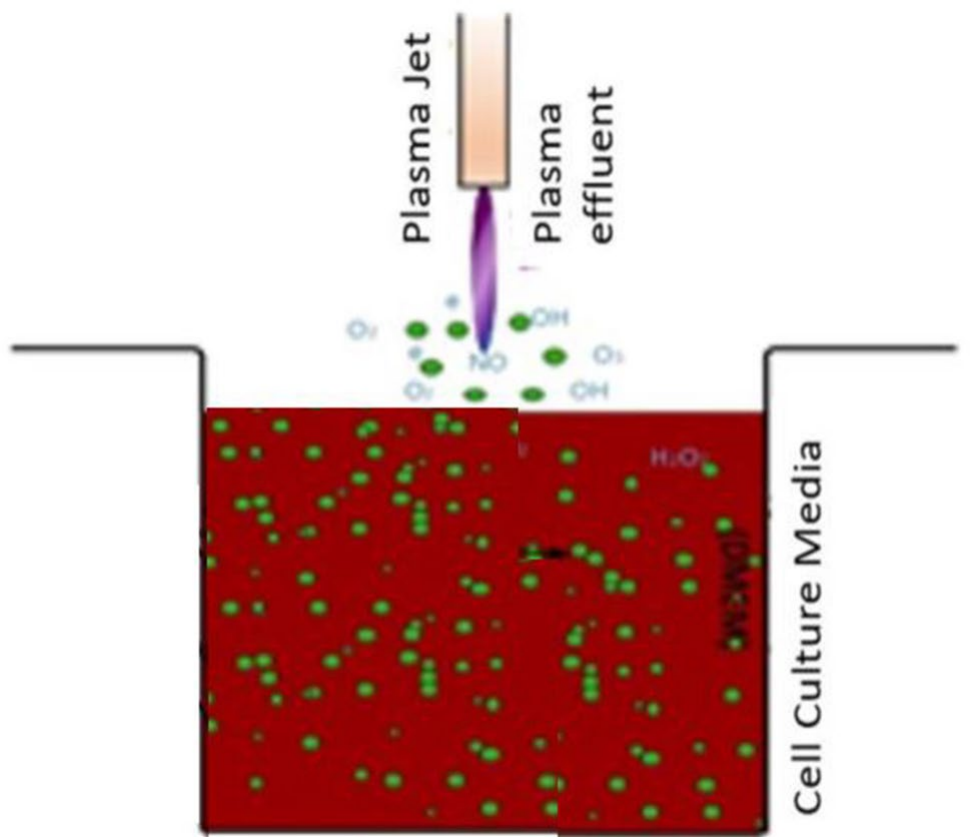
The schematic of plasma-treated cells in cell-suspension.

To evaluate cell–cell adhesion strength before and after plasma treatment, cells were treated with trypsin, collected in separate vials, and trypsin was inactivated by adding Trypsin Neutralization Solution (TNS). Two controls are adopted in this experiment. One was not exposed to gas nor plasma, while the other was treated solely with argon with no plasma ignition. For the directly-treated cases, the power was set at 20W as the low-risk level^37^ and the gas flow rate set to 1 slm. The distance between plasma plume and vials was fixed at 5 mm. Each vial was then exposed to plasma for various time intervals (30, 50, 70, 90, 100, and 120 s), as shown in Fig. 6.

After treatment, the cells of each vial were seeded on different slides to facilitate the imaging process. They were stained with a fluorescence dye (DAPI) according to cell staining protocols in “DAPI staining and fluorescence imaging”, and their images were recorded by a CCD camera attached to a Nikon fluorescence microscope (eyepiece: 10 $\times$ and objective: 40 $\times$). Use has been made of imagej cell counter plugin to count coupled and single cells. In this method, clicking in the cell image, the cell counter plugin allows to count cells. Each click adds a colored square to the cell and adds it to the tally sheet.

## Results

### De-adhesion dynamics of cells

**Figure 7:**
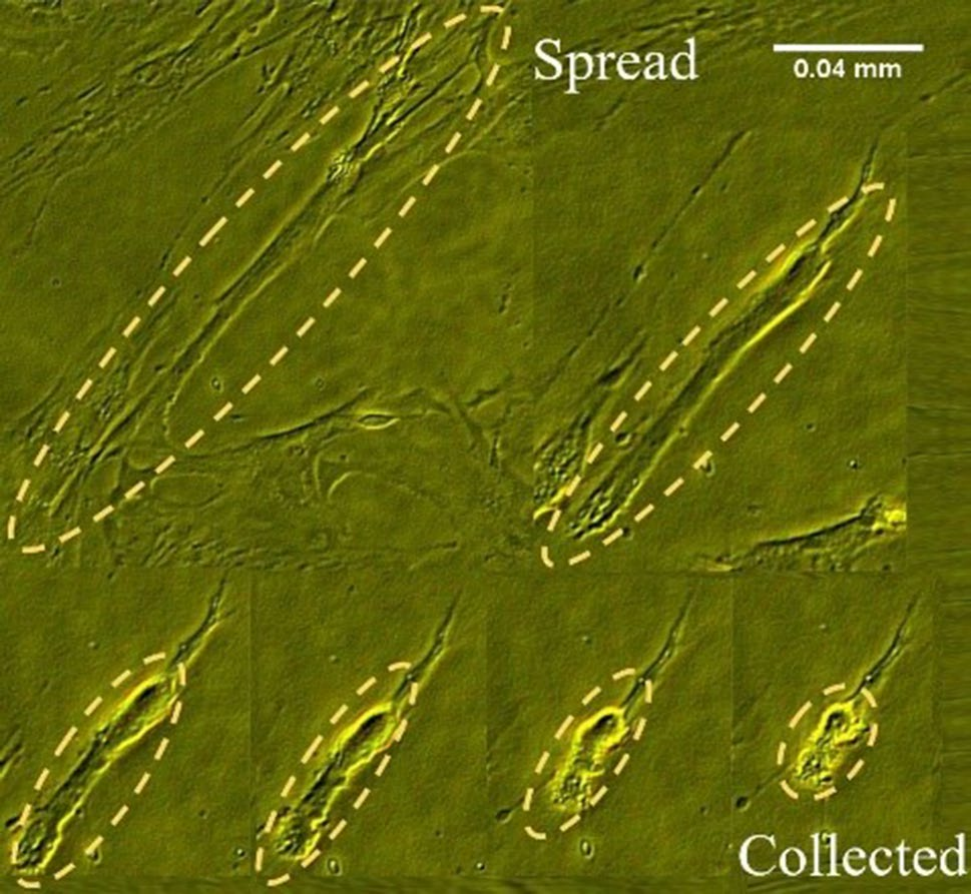
De-adhesion assay: the time-evolution process of cells during de-adhesion assay.

**Figure 8:**
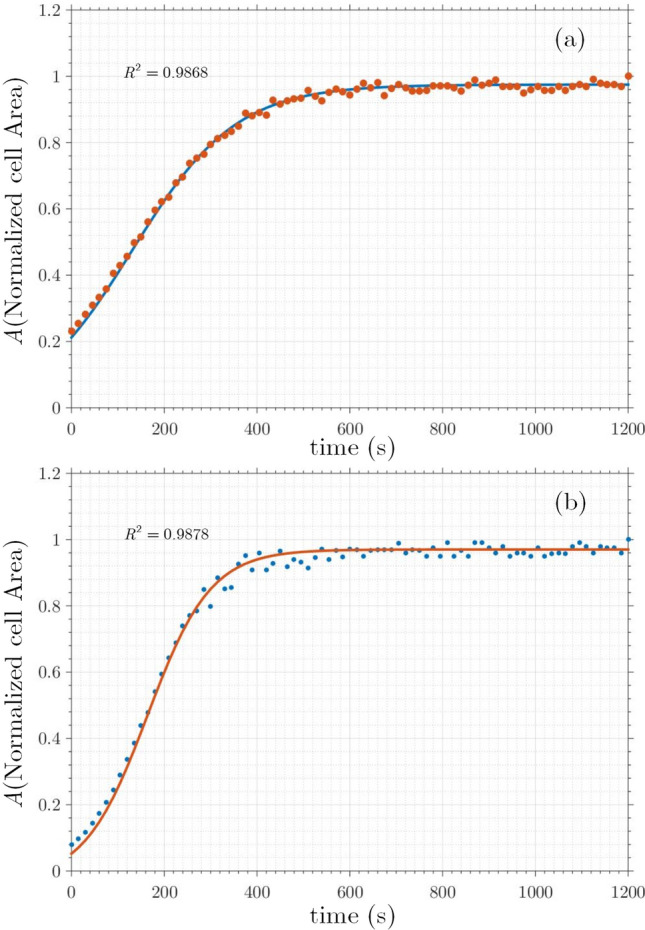
Cell normalized area vs. time. From which adhesion coefficient and elasticity were inferred (**a**) after treatment, (**b**) before treatment.

The de-adhesion test approach Sen^40^ used to determine the defined normalized cell area for both treated and non-treated cells. The outcome can be seen in Fig. 7, as can be seen the cells were contracting until they rounded up. For every 15 s the images were recorded and cell surface area was analyzed, using Imagej program and its plugins, result in the plot versus time in Fig. 8.

The cell response was sigmoid, as predicted, and was divided into three distinct stages: early delay, fast cell contraction, and saturation platform. The adhesion strength of treated cells increased in general as compared to non-treated cells. To achieve a more detailed quantitative investigation, we extract two specific time constants, $\tau _1$, and $\tau _2$. The mean time constant of $\tau _1$ for control cells is, $\tau _1$ 131.2 s, whereas it is $\tau _1$ 164 ‏s for plasma-treated cells, indicating a statistically significant difference of 20%. As these figures show, the treated cells reached saturation levels in 700 s, while control cells reached saturation levels in 500 s. More importantly, a significant and higher increase in adhesion occurred with high dosage treatment, indicating that adhesion increased in a dose-dependent manner, as reported in Table 1.

**Table 1:** The time constant and saturation time of cell exposed plasma with different generator powers, with:“Exposure Time: 60 s, Gas Flow Rate: 1 slm, and Distance between plasma plumes tip and cell: 5 mm”.

Power of plasma generator (W)	$\tau _1$(s)	Saturation time (s)
20	131.1	500
25	140.44	580
30	158.3	670
35	164	700
Cell on gel coated plate	407	950

As findings imply, the time constant and saturation time increased with increasing plasma power. For comparison, both time constant and saturation time of a cell on a gel-coated plate are also listed in the last row.

According to Keidar^36^, Weltmann, Haertel^32,33,43^ and etc. (demonstrated in separate papers), increasing the adhesion of the cell-substrate after plasma treatment attributed to increased integrin protein experience and activity, which leads to improved cell-substrate adhesion.

### Focal adhesion assay results

According to the previous section, cell adhesion increased by 1.4 after plasma treatment. The increase in cell-focal adhesion was measured using a straightforward technique in this study. Cells were prepared and treated directly with plasma (for different exposure time of 30, 50, 70, 90, 100, and 120 s) as described in “Focal adhesion”. Using a CCD coupled to a Nikon Microscope, the images were recorded and combining CLAHE and LOG-3D plugins facilitated FA quantitative evaluation.

**Figure 9:**
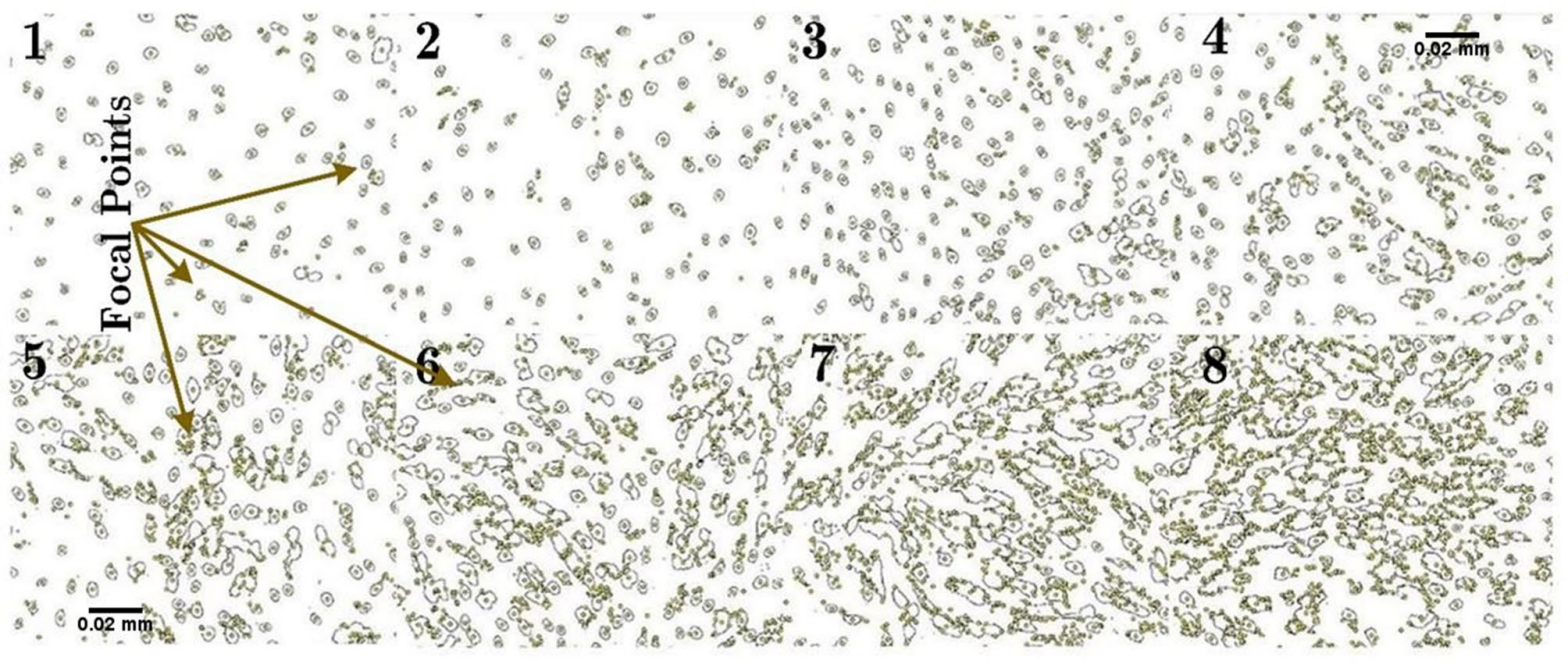
Focal adhesion images for: (1) before treatment and before FA formation, (2) cells treated with Argon (plasma-off) after the formation of FA, and from 3 to 8 the cells were directly exposed to plasma (power = 20 W) for different exposure times: (3) t = 30 s, (4) t = 50 s, (5) t = 70 s, (6) t = 90 s, (7) t = 100 s, (8) t = 120 s. As can be seen in 3–8, more focal points are formed. Increase in FA points depends on the exposure time.

The results, shown in Fig. 9, confirmed the increase in focal points in a dose-dependent manner during the plasma exposure. When the exposure time was between 70 and 120 s, the focal points increased by 1.3–1.7, respectively, which is consistent with the results of the previous section.

### Shear stress assay results

No significant difference in physical resistance to shear stress between treated and untreated cells at gas flow rates between 1 and 1.2 slm and culture-media flow rates between 15 and 45 dyne/m$^2$ was measured. The critical shear stresses of untreated and treated stem cells were measured as 25.42 and 25.57, respectively. The student’s t-test was used in statistical analysis and p0.05 was considered significant. All data were expressed as mean SEM from at least three independent experiments, in which more than 580 cells were counted. Therefore, the amount of gas flow has no effect on cell separation in this range of the flow rate.

### Cell–Cell adhesion strength results

**Figure 10:**
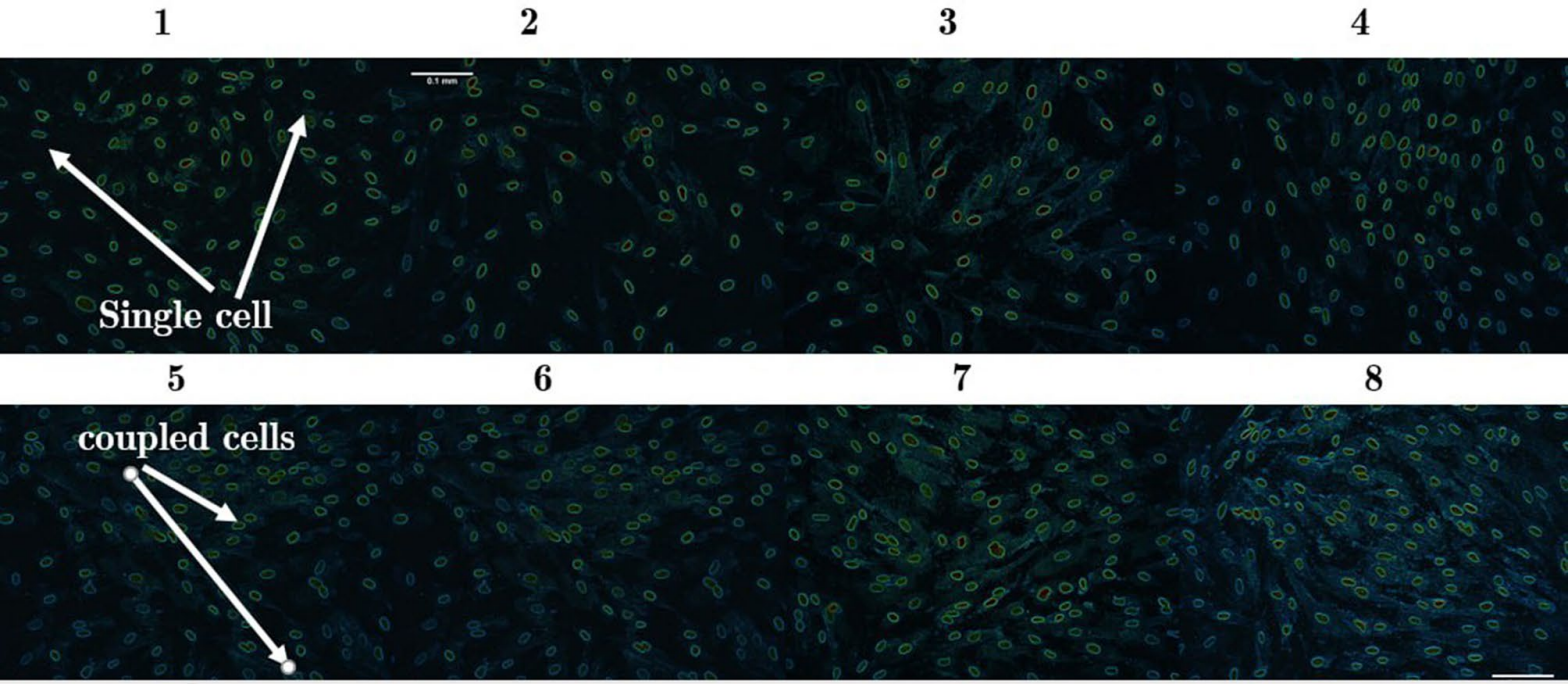
Cell images in cell–cell adhesion assays (1) before treatment, (2) cells treated with Argon (plasma-off). Almost all cells are single. For 3–8, cells were directly exposed to plasma (power = 20 W) with different exposure times: (3) t = 30 s, (4) t = 50 s, (5) t = 70 s, (6) t = 90 s, (7) t = 100 s, (8) t = 120 s. As can be seen in 3–8, more couples are formed after treatment and the number of couples increases depending on the exposure time.

The experiment was conducted on two groups of control cells: the first group was not exposed to either gas or plasma, while the second group was given simply argon treatment, that is, no plasma ignition, see Fig. 10(1, 2). For the treated cells, the power was set at 20W as the low-risk level^37^, as documented in Fig.10(3–8) for increasing exposure time ranging from 30 to 120 s.

**Table 2:** The ratio of doublets to singles before and after treatment.

	Control	Plasma-off	$t=$30 s	$t=50$s	$t=70$s	$t=90$s	$t=100$s	$t=120$s
Mean of ${N_{2}}/{N_{1}}$	0.097	0.113	0.318	0.400	0.509	0.581	0.597	0.648

As can be seen, this ratio increases after treatment, the longer the exposure time, the higher the ratio.

As shown in Fig. 10(3–8) as well as in Table 2, the ratio of doublet to singlet increased when the cells were exposed to plasma. This ratio is larger for longer exposure times, as can be shown. Therefore, it can be inferred that following treatment, cell–cell adhesion increased. The increase in cell adhesion could be a consequence of the increase in VDW force between cell–cell.

**Figure 11:**
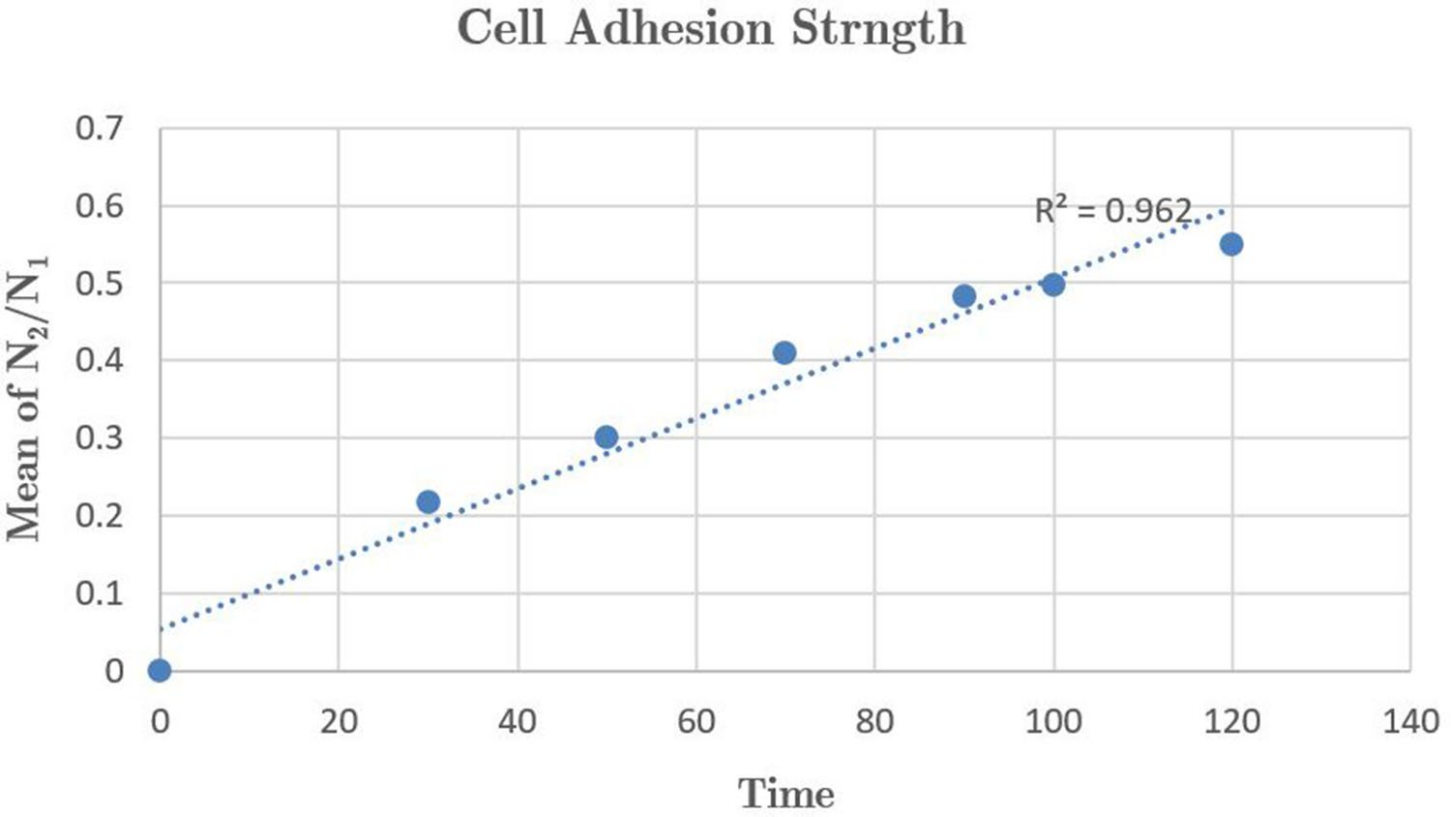
When the cells were exposed to plasma, the ratio of doublet (N$_2$) to singlet (N$_1$) increased.

Given that the number of cells sticking to each other following plasma exposure is significantly larger than single cells, the ratio of binary cells to single cells in terms of cell volume percentage could be a straight line passing through the origin which gradient of that is the adhesion strength, as can be seen in Fig. 11.

To explain that, we borrow a theoretical formula by Stainton^23^ to predict the number of doublets that would form in a cell suspension after treatment. Sainton showed that the ratio of doublets to singles, $N_2/N_1$, is proportional to the volume fraction phi of cells as

1 $$\begin{aligned} \dfrac{NN_2}{N_1} =4\varphi \left( \lambda ^3-1 \right) \exp \left( \frac{\varepsilon }{kT} \right) \approx \dfrac{N_2}{N_1} \end{aligned}$$

where $\lambda$ and $\varepsilon$ are the potential well parameters, $\varphi$ is the cell fraction volume, and *N* is the total number of cells. Therefore, the graph of the $N_2/N_1$ versus cell volume fraction should be a straight line passing through the origin. The slope of this line is the adhesion-resistance coefficient^23^.

## Discussion of the first set of results

As detailed in the previous result sections, the role of the plasma on the cell–ECM adhesion and the cell–cell adhesion strengths have been investigated in the different experiment set-ups by employing the de-adhesion assay and focal adhesion analysis. Analyzing the cell–ECM adhesion for the plasma treated and untreated cases show that the cell–ECM adhesion increases for the plasma treated cases by the dose and the time exposure attributed to the enhancement of the production and activity of the integrin protein in the medium. Studying the plasma role on the cell–cell adhesion by ignoring the activation of the integrin protein by creating a suspension shows that plasma increases the cell–cell adhesion with increasing the number of coupled cells in comparison to untreated cases. We claim that, in this scenario, the probable rising of the adhesion cell could be related to the increasing of Van der Waals (VDW) force rather than integrin production and activation^23,26^. More dipoles developed when the cells are exposed to plasma, leading in an increase in VDW force. To find the physical reason of this observation, it should be considered that the cell surface is the first part to be exposed to the plasma-induced electric field and RONS. The cell wall itself contains large amounts of polysaccharides and other natural polymers. As a result, positive ions are adsorbed from the media to the surface^44,45^. Carstensen et al. showed that the cell wall is actually an ion exchanger^46,47^. In fact, charged groups on the cell wall exchange small molecules with the environment^45,48^. Therefore, it is expected that the electrical properties of the wall will change with the concentration of ions in the environment^48^. In plasma-exposed media, a large number of ions are generated, which might change the charge distribution in this area. Although the conductivity of the cell wall remains constant at low ion concentrations, it rapidly rises as the ion concentration increases^46,47^. Consequently, the presence of plasma ions alters the electrical properties of the wall, including its conductivity and permittivity. The dielectric constant of any substance is affected by the polarization of the molecules. In the case of non-polar molecules, polarizability results from electronic polarization, that is, an electron is shifted relative to the nucleus. Accordingly, to comprehensively study the effect of plasma-induced fields, with the source frequency in the MHz range (as in our case), the rate of increase in plasma-induced dipole formation as well as electrical conductivity, the dielectric permittivity of the cell wall, and dielectric permittivity of the surrounding aqueous media must be taken into account. All of these parameters have major roles on the intercellular van der Waals force as a key phenomenon in increasing the cell–cell adhesion after plasma treatment. In the next section, a theoretical model is adopted to demonstrate the effect of each of the parameters involved in plasma therapy, including electromagnetic waves and charged species, on the intercellular van der Waals force. In this regard, the role of these parameters on the dipole creation and changing the dielectric constant of the culture medium, attributed on the Hammaker coefficient modification, is investigated semi-empirically.

## Dipole formation (van der Waals Force)

In the previous section, it was discovered experimentally that exposing a cell to plasma increases the cell–cell adhesion. Generally speaking, according to Curtis^49^, the state of the plasma-lemma lipids, which is influenced by physical plasma, affects cell adhesion. These effects can be explained either in terms of the van der Waals, VDW, forces or changes in surface fluidity, both of which are influenced by cold atmospheric plasma. Therefore, to study the plasma-induced cell-adhesion changes, the VDW forces must be considered before and after plasma treatment. The VDW force, an ever-present force among all molecules, plays a valuable role in cell adhesion. VDW force caused by the fluctuation of electric dipole-moment of molecules is a ubiquitous force between two molecules. Here, charge fluctuations occur for two reasons: (1) The presence of a plasma-induced electric field during plasma exposure, (2) The proximity of two molecules with a total zero charge. This force has a meaningful influence on various phenomena, including cell adhesion, metastasis, and fusion^26^.

**Figure 12:**
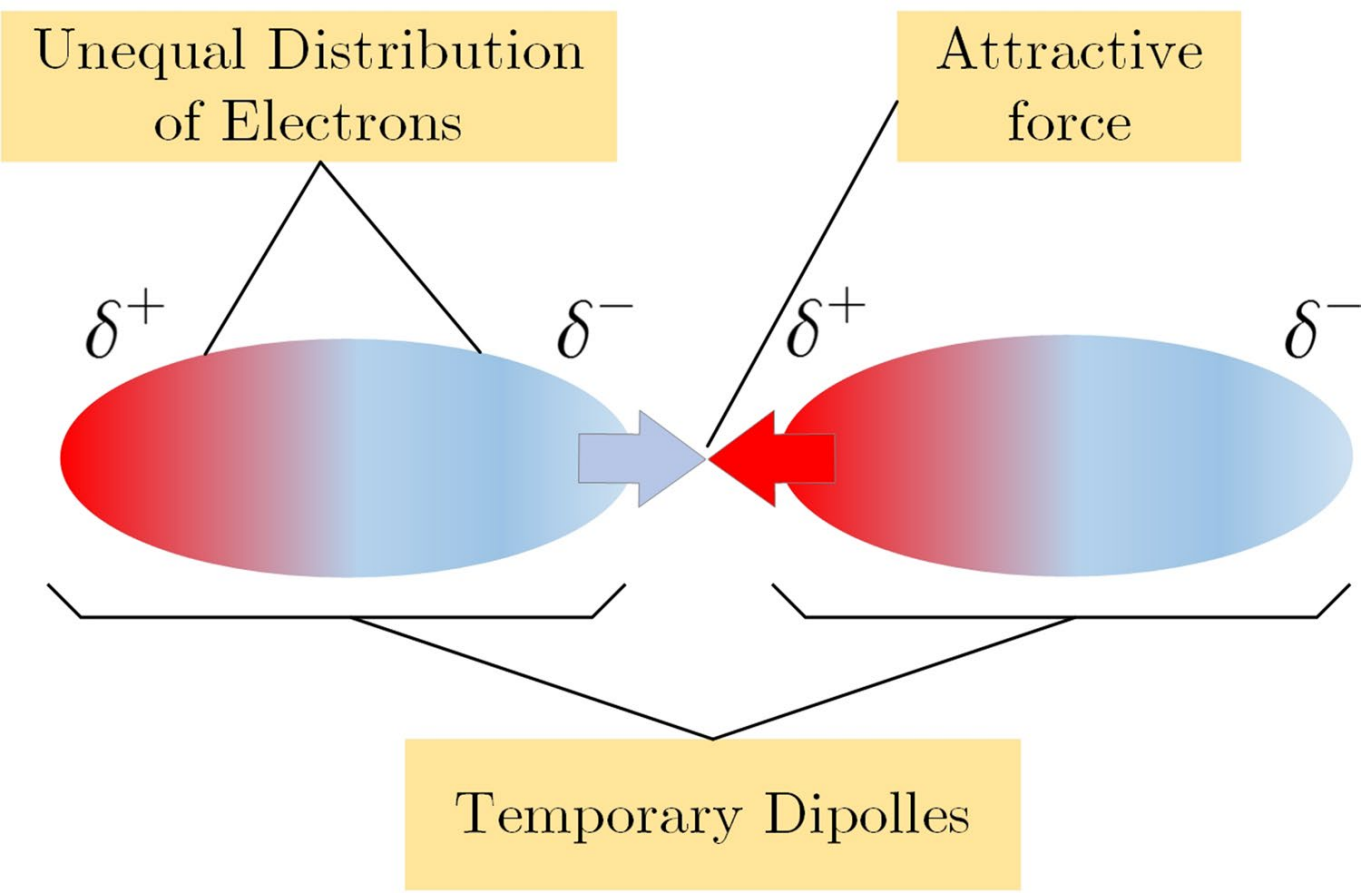
A schematic of the VDW force between temporary dipoles.

Putting an isotropic particle in a homogeneous environment with a uniform electric field, the electric field surrounding the particle is disrupted^27,50^. Charge separation occurs at the particle surface as a result of the electric field, leading in the production of transitory electric dipoles. A similar scenario might occur when a cell is exposed to atmospheric plasma, as shown schematically in Fig. 12.

### Exposed electromagnetic field effect on VDW force (dipoles creation)

Regarding the fact that most human cells are spherical with a radius of 10–100 $\upmu$m, the size of a human cell and the wavelength of the exposed electromagnetic wave are comparable as long as the frequency is about 10 THz. As a result, the quasi-static approximation could be used for the frequencies of relevance in this work, 13.56 MHz. To figure out the solution of the electric field, use has been made of the Maxwell’s Equations and considering that $\nabla \times \nabla \Psi =0$ for all $\Psi$, the electric field can be written in terms of the scalar potential of $\varphi (r)$:

2 $$\begin{aligned} E(r) = -\nabla \varphi (r). \end{aligned}$$

At the first approximation, without considering the role of the charged plasma species in the medium on one hand and using the assumption of $\rho =0$ for biological cells in the scale under study on other hand, we can write Laplace equation as:

3 $$\begin{aligned} \nabla ^2\varphi (r) = 0. \end{aligned}$$

**Figure 13:**
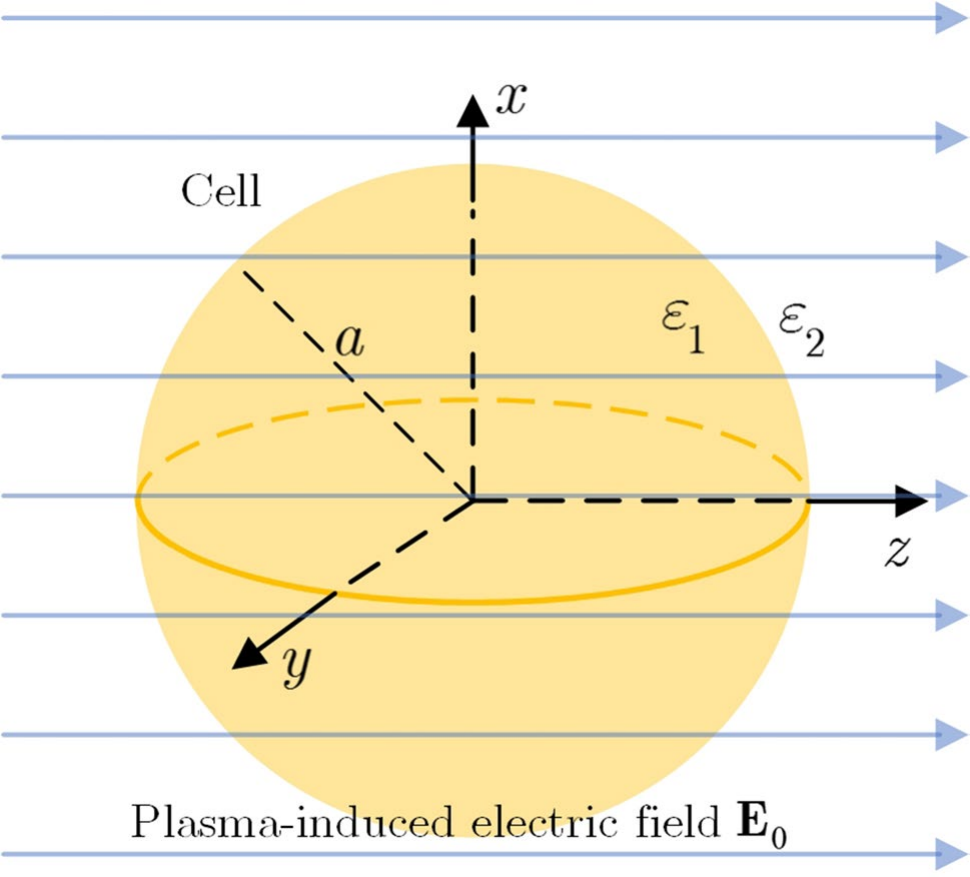
Plasma-induced electric field, ${\textbf {E}}_0$, applied to a spherical cell with no organelles, the radius of cell is *a*.

Laplace Equation is solved by considering a cell as a dielectric sphere of radius *a* with its center at the origin of the coordinates and a constant and uniform electric field in *z*-direction, ${\textbf {E}}_0 = E_0\hat{z}$, see Fig. 13.

The solution is:

4 $$\begin{aligned} \varPhi (r, \theta , \varphi ) = \dfrac{U(r)}{r}P(\theta )Q(\varphi ), \end{aligned}$$

According to Eq. (2), the incident potential is:

5 $$\begin{aligned} \varPhi (r) = -E_0z, \end{aligned}$$

Therefore,

6 $$\begin{aligned} \varPhi (r) = -E_0r\cos \varphi =-E_0rP_1(\cos \theta ), \end{aligned}$$

here $P_1(\cos \theta )$ is the Legendre polynomial of order $n=1$. Therefore, the total potential outside of the cell is $\varPhi (r, \theta ) = \varPhi _0(r, \theta )$.

In this equation, the electric potential at infinity must be equal to the initial potential and also, as a good assumption, the electric potential at the origin of the coordinates could be limited due to lack of charge inside the cell.

Accordingly, we can write:

7 $$\begin{aligned} \varPhi _\text {in}(r, \theta ) =\sum _{n=0}^{\infty } A_nr^nP_n(\cos \theta ), \end{aligned}$$

and

8 $$\begin{aligned} \varPhi _\text {out}(r, \theta ) =\sum _{n=0}^{\infty } B_nr^{-(n+1)}P_n(\cos \theta ) -E_{0}rP_1(\cos \theta ). \end{aligned}$$

By applying the boundary conditions such that the electric potential at the cell surface is continuous $\varPhi _\text {in}(r=a, \theta ) =\varPhi _\text {out}(r=a, \theta )$, and the vertical component of the flux density is constant, we will have:

9 $$\begin{aligned} -E_{0}rP_1(\cos \theta ) =\sum _{n=0}^{\infty }\left[ A_nr^n + B_nr^{-(n+1)}\right] P_n(\cos \theta ), \end{aligned}$$

10 $$\begin{aligned} -\varepsilon _0 E_{0}aP_1(\cos \theta ) =\sum _{n=0}^{\infty }\left[ \varepsilon _1 A_nnr^{n-1} + \varepsilon _2 B_n(n+2)r^{-(n+2)}\right] P_n(\cos \theta ). \end{aligned}$$

Considering that the Legendre polynomials are orthogonal, the terms in the right-hand side of Eqs. (9) and (10) are eliminated and only $n=1$ is not vanished. Accordingly:

11 $$\begin{aligned} \varPhi _\text {in}(r, \theta ) = -\dfrac{3\varepsilon _1}{\varepsilon _1+2\varepsilon _2}E_0r\cos \theta \end{aligned}$$

12 $$\begin{aligned} \varPhi _\text {out}(r, \theta ) = -\dfrac{\varepsilon _1-\varepsilon _2}{\varepsilon _1+2\varepsilon _2}E_0\dfrac{a^3}{r^2}\cos \theta -E_0r\cos \theta \end{aligned}$$

Making use of Eq. (2), the electric field can be obtained from Eq. (11) by changing the coordinates to rectangular. So we will have:

13 $$\begin{aligned} {\textbf {E}}_\text {in}(r, \theta ) = \dfrac{3\varepsilon _1}{\varepsilon _1+2\varepsilon _2}E_0\hat{z}, \end{aligned}$$

the cell is an electric dipole of which its moment can be interpreted as the volume integral of the polarization ${\textbf {P}}$, therefore,

14 $$\begin{aligned} P = (\varepsilon _1-\varepsilon _2)E =3\varepsilon _1 \dfrac{\varepsilon _1-\varepsilon _2}{\varepsilon _1+2\varepsilon _2}E_0, \end{aligned}$$

and hence the dipole moment is:

15 $$\begin{aligned} {\textbf {P}} =4\pi \varepsilon _1\left( \dfrac{\varepsilon _1-\varepsilon _2}{\varepsilon _1+2\varepsilon _2}\right) a^3E_0\hat{z}, \end{aligned}$$

Consequently, the cell behaves as an electrical dipole with the dipole moment of Eq. (15). Thus, two close cells behave like a dipole which is shown in Fig. 10 schematically. Subsequently, the VDW force will change between cells, interpreted by Eq. (4).

**Figure 14:**
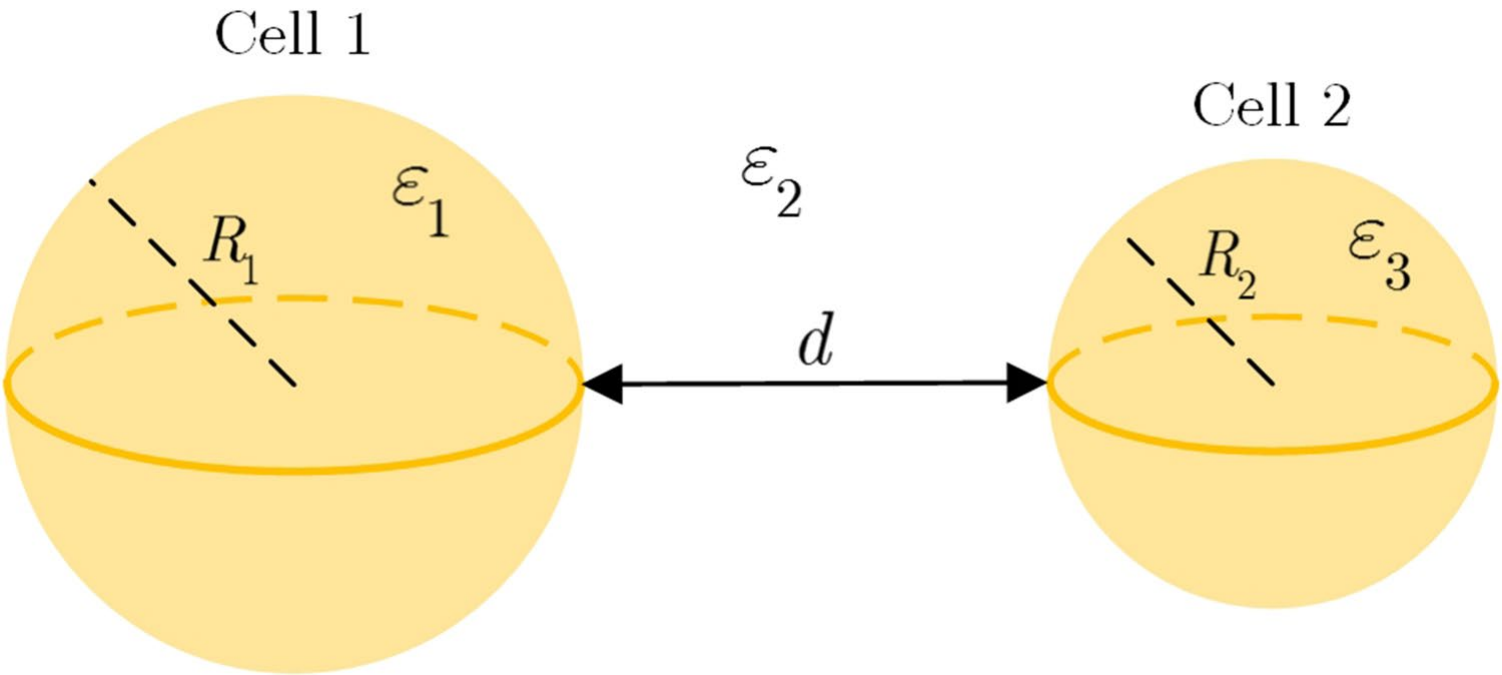
The schematic of cells, their distance, media and their permitivities.

### Charged plasma species effect on VDW force

At the previous sub-section, the role of the exposed electromagnetic field in plasma therapy had been investigated on creation of dipole in the culture medium and so on VDW force between the cells related to cell–cell adhesion. At this part, the role of the delivered charge in the medium during the plasma therapy will be investigated on the VDW force between the cell and so on the cell–cell adhesion.

To this end, the cells are considered as sphere with no organelles inside, for simplicity. Therefore, the VDW energy of interaction depends on the surface geometry. For two spherical objects it is expressed as:

16 $$\begin{aligned} G = -\dfrac{A}{6d} \left( \dfrac{R_1R_2}{R_1+R_2}\right) \end{aligned}$$

where *d* is the separation of two cells, *A* is Hammaker coefficient, and $R_1$ and $R_2$ are the radius of two cells which are assumed equal in this study (see Fig. 14). Therefore, *G* can be written as:

17 $$\begin{aligned} G = -\dfrac{A}{6d} \left( \dfrac{R_1^2}{2R_1}\right) = -\dfrac{A}{6d} \left( \dfrac{R_1}{2}\right) \end{aligned}$$

According to Hammaker ^51^, A is given by:

18 $$\begin{aligned} A = \frac{3}{4}kT \dfrac{(\varepsilon _1-\varepsilon _2)(\varepsilon _3-\varepsilon _2)}{(\varepsilon _1+\varepsilon _2)(\varepsilon _3+\varepsilon _2)}, \end{aligned}$$

in which *k* is Boltzmann constant, *T* is the temperature, and $\varepsilon _1$, $\varepsilon _3$, and $\varepsilon _2$ are the dielectric constants of cell 1, cell 2, and the cell culture medium, respectively. In this study $\varepsilon _3 = \varepsilon _1$, therefore:

19 $$\begin{aligned} A = \frac{3}{4}kT \dfrac{(\varepsilon _1-\varepsilon _2)^2}{(\varepsilon _1+\varepsilon _2)^2}. \end{aligned}$$

The VDWF is:

20 $$\begin{aligned} G = -\dfrac{A}{6D^2} \left( \dfrac{R_1}{2}\right) . \end{aligned}$$

So, to find the effect of the delivered charge in the plasma therapy on the VDW force between the cells, we have to follow the change of the Hammaker Coefficient due to presence of these species in the culture medium. When cells and culture media are exposed to the plasma their electrical conductivity changes, as a result of the conductivity modification. In this sense, the relative electrical permittivity, $\varepsilon _1$, $\varepsilon _2$, will also change and eventually, it results in a change in Hammaker coefficient. Considering the water and cell wall permittivity, in Eq. (19), for example 80 and 60, respectively, it is found that a 2% decrease in the permittivity of the cell wall leads to a rise of 13.4% in the Hammaker coefficient, provided that the permittivity of the aquatic media remains constant. A further decrease in the permittivity of this wall leads to a sharp increase of 84.5% in the Hammaker coefficient. On the other hand, if the permittivity of the aqueous media surrounding the cell reduces by 40%, the Hammaker coefficient significantly increases, 19.6-times.

**Figure 15:**
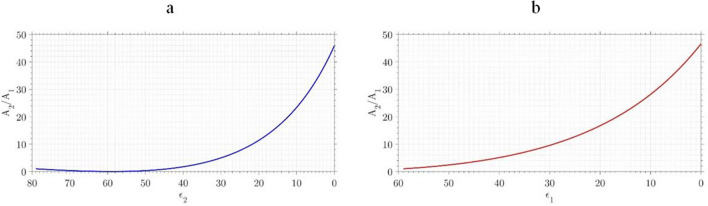
The ratio of $A_2/A_1$ increases by reduction in (**a**) media permittivity, (**b**) cell permittivity.

In Fig. 15a,b, the parameter $A_2/A_1$ ($A_1$ and $A_2$ are the Hammaker coefficients before and after treatment, respectively) vs. the value of the media permittivity and cell-wall permittivity has been illustrated, respectively.

**Figure 16:**
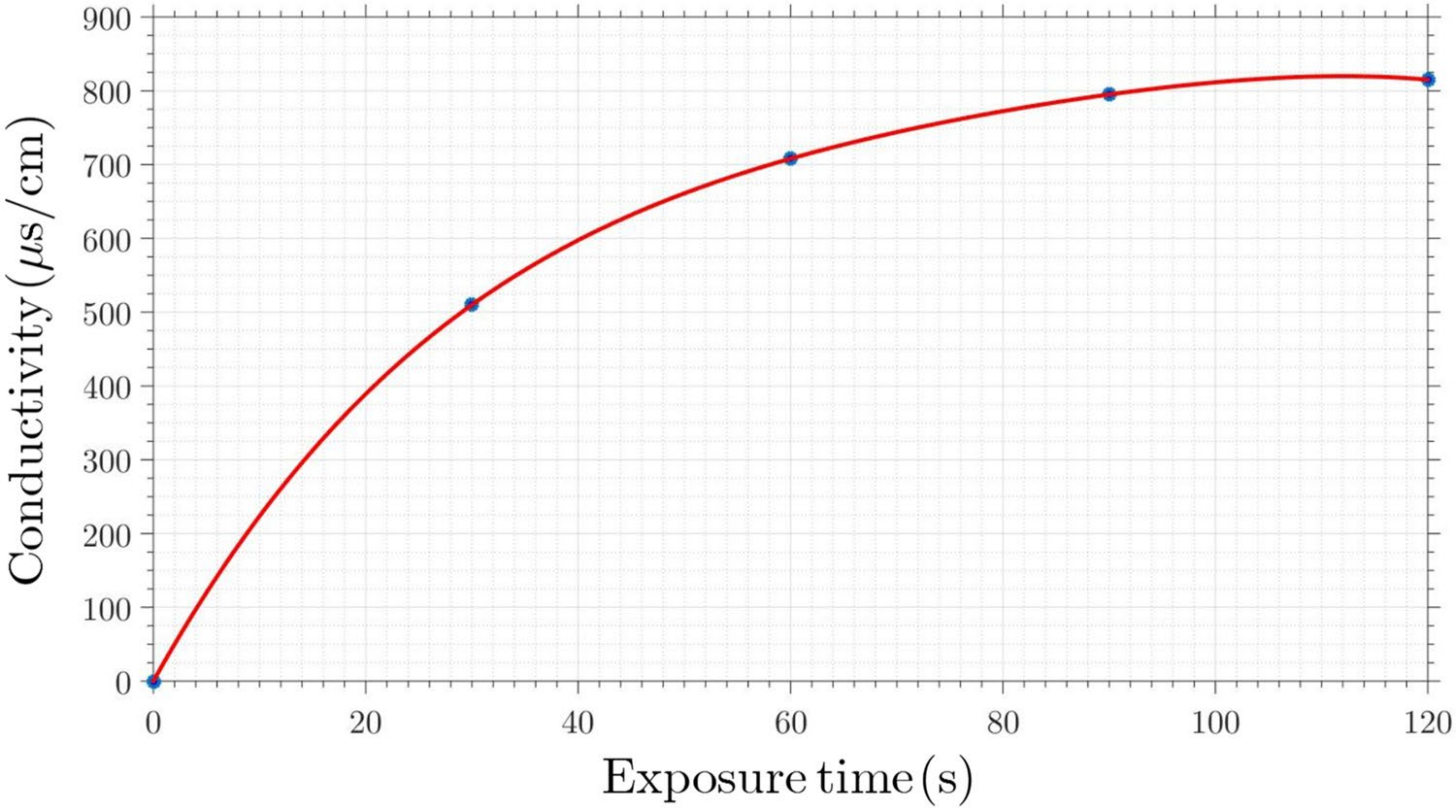
Conductivity of medium vs. the plasma exposure time.

As can be seen in this figure, when the permittivity of the cell-wall decreases the ratio $A_2/A_1$ increases significantly. On the other hand, by reducing the permittivity of the media, until the permittivity of the media is 40% of its initial value after that, this ratio increases significantly. Regarding the fact that the VDW force directly depends on the Hammaker coefficient, an increase in this coefficient causes a rise in the VDW force. To determine the changes in Hammaker coefficient during the plasma therapy, *A*, the conductivity of the media was measured in 6 vessels holding deionized water and exposed to plasma at different exposure times (between 30 and 120 s). These measurements are documented in Fig. 16. As seen in this figure, the plasma treatment greatly increases the PAW’s conductivity by adding more charged plasma species to the environment. The electrical conductivity provides information on concentration of ions present in aqueous media.

As can be seen in Fig. 16, electrical conductivity increases from $0\pm 10$ to $723\pm 10\,\, \upmu$S/cm for the first 60 s plasma treatment, then it reaches to a saturation plateau from $60-120$s exposure time. Linear regression of the first part gives following equation ($R_2=0.99$):

21 $$\begin{aligned} \sigma = 591.20+4.74\tau . \end{aligned}$$

Variation of $\sigma _{\text {PWA}}$ results from charged species production and consumption mechanisms occurring in the liquid phase, resulted from a complex interaction between the plasma and liquid phase which includes; a) the diffusion of gaseous species from the plasma to the liquid bulk, b) the stimulation of the liquid interface giving rise to new species in the sub-interface layer and then to their in-depth diffusion and, c) PAW heating effect induced by the plasma source.

At the last step, we try to find the charged plasma species effect on the dipole formation. How electrical charge is distributed near the cell membrane is the key to many of the problems associated with cells interacting with external electric fields. Many papers have investigated the spatial distribution of charge near the surface of biological membranes. The permittivity of the cell in an aqueous media relates to media permittivity by Maxwell- equation.

Since the generation of a dipole in a cell at a given frequency depends on the conductivity of the surrounding medium, the increase in the conductivity affects polarization too. Considering Eq. (15), the term in bracket will be changed by conductivity modification as:

22 $$\begin{aligned} P(z) \propto \left( \dfrac{\varepsilon _1-\varepsilon _2}{\varepsilon _1+2\varepsilon _2} \right) , \end{aligned}$$

**Figure 17:**
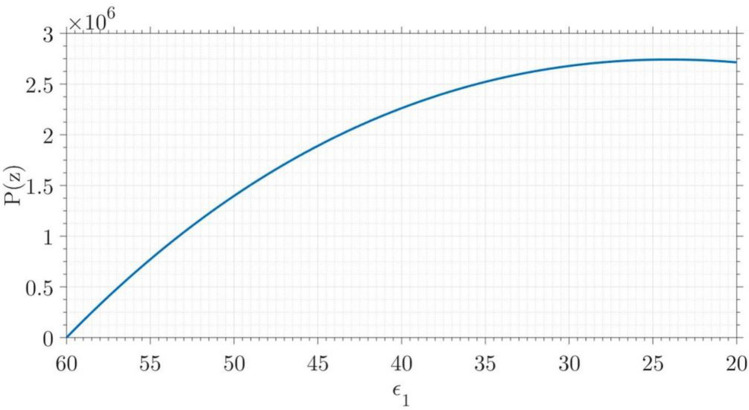
Polarization moment vs. the permittivity of the cells.

As shown in Fig. 17, as it was expected, the decrement of permittivity of the cell wall results in enhancing of the dipole moment.

### Temperature and cell surface roughness effects on VDW force

Due to the slight changes in the temperature of the exposed culture media, the dielectric constant dependence on temperature is not considered in the theoretical model. Another parameter affecting the VDW force is the cells surface-roughness. The smoother the cell surface, the higher the VDW force^26^. Moreover, reducing the surface roughness decreases the closest-distance between the cells and so, the cells get closer to each other to experience the higher VDW force. The qualitative study of the plasma therapy effect on the VDW force between the cells (equivalently, cell–cell adhesion) through changing the cell roughness has been investigated empirically.

**Figure 18:**
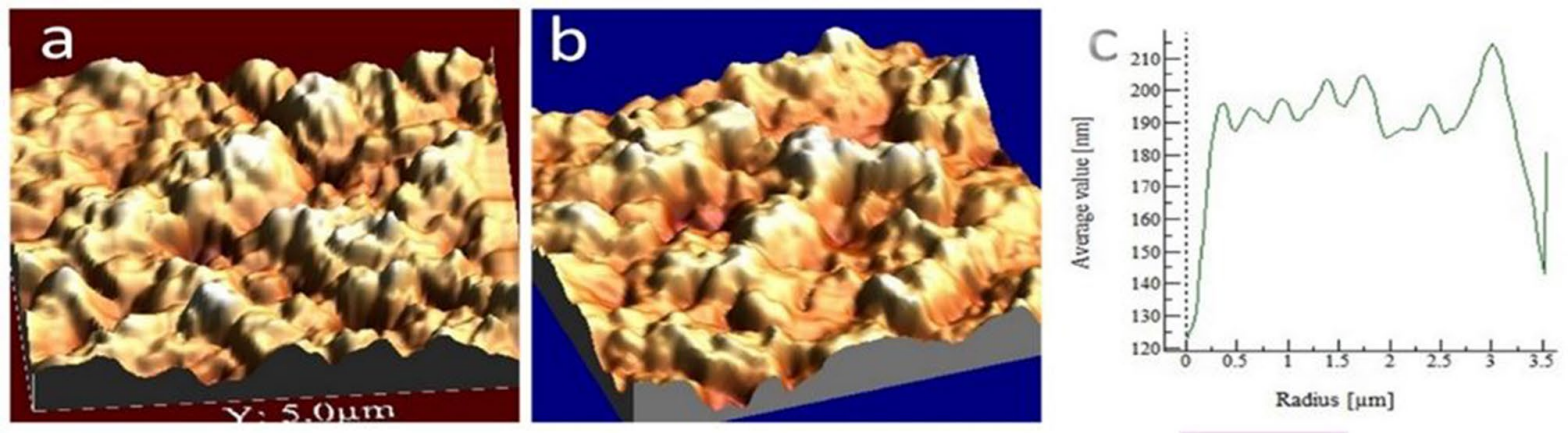
Cell surface roughness, (**a**) before plasma treatment, (**b**) after plasma treatment, (**c**) mean height.

The roughness of cell surface can also affect the VDW force. In this regard, we used Atomic Force Microscope, AFM (Park Scientific Instruments-Cp Auto Probe (LFM, Contact AFM)) images to evaluate the changes in cell roughness after plasma treatment. The images of cell roughness before and after treatment, Fig. 18a,b, show a softer cell surface after treatment.

To characterize the surface topography, the amplitude parameters are the principal parameters. The average roughness ($R_a$) and the root mean square roughness ($R_q$) were used to determine roughness of the cell before and after treatment.

**Table:** 

Arithmetic average height $\overline{Z}$, a general description of height variations.	$\overline{Z}(N, M) = \frac{1}{N} \sum _{x=1}^{N} Z(x, y)$
Average roughness ($R_a$), gives the deviation in height.	$R_a = \frac{1}{N} \sum _{x=1}^{N}\left( Z(x, y) - \overline{Z}(N, M) \right)$
Root Mean Square (RMS) roughness ($R_q$) represents the standard deviation of surface heights.	$R_q(N, M) = \sqrt{\frac{1}{N} \sum _{x=1}^{N}\left( Z(x, y) - \overline{Z}(N, M) \right) ^2}$

The results show in Table 3.

**Table 3:** Nano-scale surface height change: transverse profile in terms of cell surface roughness.

	$R_{pv}$	$R_q$	$R_a$	Mean height
Non-treated stem cell	336.95	44.8525	35.6375	183
Treated stem cell	313.2	40.005	31.2025	166
Non-treated stem cell	336.95	37.03	28.65	196.3
Treated stem cell	211.1	29.14	22.64	114.3
Non-treated stem cell	383.50	46.86	44.15	206
Treated stem cell	368.9	54.38	36.91	185.7
Non-treated stem cell	361.7	47.19	36.61	193.5
Treated stem cell	255.7	35.52	28.16	122.7
Non-treated stem cell	366.6	51.45	40.40	221.2
Treated stem cell	296.5	38.06	29.84	157.5

In the Table 2, $R_a$ is the average roughness, $R_q$ is the root mean square of roughness and $R_{\text {pv}}$ is the maximum valley depth ($R_v$) to maximum peak height ($R_p$) in nm scale.

To make sure that the $R_q$ values are making sense, the height points need to be uniformly distributed according to a normal distribution. AFM analysis of both non-treated and plasma-treated cells indicated a topographic change in surface roughness at the nanometer scale during the plasma therapy. The uniformity of the topographic features of the stem cell surface increased after plasma exposure, as shown in Fig. 18, in which the topography of the stem cells surface at the nanometer scale is showed. The transverse profile calculated with respect to the roughness data confirmed the nano-scale change in surface height (Table 3). Compared to non-treated cells, the topographic image of the plasma-treated cell in Fig. 18b showed a more uniform surface characteristic. Peak heights of max 110-200nm observed that were 10% lower than those observed in non-treated cells (125–225 nm). These results showed that the roughness of the non-treated cell surface is almost 10% more (at the nanometer scale) than the treated cell. Consequently, given that the VDW force is inversely proportional to the distance between the two cells, it can be deduced that a 10% decrease in the distance between the two cells will increase the VDW force by up to around 20%. This increase is simply due to the reduction in the gap between the two cells. For now, we have ignored the rise in VDW forces due to the smoothing of the cell surface. This amount is added to that of adhesion increased due to dipole formation and Hammaker coefficient change.

## Conclusions

The adhesion of the stem cells appears to be a key factor in determining their differentiation and fate. In this research, de-adhesion assays were utilized to evaluate stem cell adhesion after plasma treatment. Using this method, it was revealed that cell–ECM and cell–cell adhesions increased in a dose-dependent manner after plasma treatment. Experimental studies indicated an increase in the cell adhesion coefficient, for which two significant reasons were evoked following plasma treatment; (1) the improvement of integrin expression and activation which influence the cell–ECM adhesion and (2) the increasing of VDW force which increases the cell–cell adhesion. In this regard, in investigating the cell–cell adhesion, the raise in VDW force obtained in suspension condition, to ignore the role of the integrin proteins, was due to three reasons; (1) the creation of electrical dipoles, (2) changing the dielectric constants of the culture medium and the cell-wall and, (3) decrement in the cell surface roughness. The rise in van der Waals force can be explained by an increase in dipole formation as a result of plasma treatment. Moreover, cell polarization was investigated both theoretically and experimentally, after plasma therapy. It was also discovered that plasma treatment enhances the Hammaker coefficient. Increasing in this coefficient indicates an increase in cell conductivity, which was evaluated in the current study. In accordance with the foregoing findings, it was revealed that the cell’s surface had been modified as a result of being exposed to plasma. A change in the roughness of the cell surface might also explain the increase in van der Waals force. Taking all of the findings into account, it was revealed that the intercellular van der Waals force rises, as does the stem cell’s adhesion. A change in the roughness of the cell surface might also explain the increase in van der Waals force. Taking into consideration all of the data, it was revealed that the intercellular van der Waals force, as well as the stem cell’s adhesion, increases.

## Supplementary Information

Supplementary Information.

## Data Availability

The datasets generated during and/or analysed during the current study are available from the corresponding author on reasonable request.
