# Supplementary material for: A van der Waals force-based adhesion study of stem cells exposed to cold atmospheric plasma jets
Source: Sci Rep. 2022 Jul 15;12:12069. doi: 10.1038/s41598-022-16277-1 (PMC9287354; doi:10.1038/s41598-022-16277-1)
Supplement: Supplementary file 1 — Supplementary Information. [file 41598_2022_16277_MOESM1_ESM.docx]

**Supplementary material**

1. Flow Cytometry data of the Human Bone Marrow Mesenchymal Stem Cells:

The stem cells used in the present work have not been directly taken by our team and provided by the institution of **Stem Cell Technology Research Center.** In the following, one can find the Flow Cytometry data and results of the Human Bone Marrow Mesenchymal Stem Cells studied in this paper. Stem Cells and the related Flow cytometry results have been provided by **Stem Cell Technology Research Center (STRC), Tehran, Iran (Special thanks to Dr. Seyed Ehsan Enderami).**

**Method:**

**Characterization of Mesenchymal stem cells using flow cytometry:**

Human bone marrow was used to extract mesenchymal stem cells, which were then grown in a medium containing DMEM/F12 and 10% FBS. Every three to four days, cells experienced a passage. During passage 3, cells were detached using trypsin-EDTA, divided into six tubes with a total of around 5x10^5^ single cells suspended in DMEM, and centrifuged for five minutes at 1200 RPM. Then, 1ml of human serum was used to re-suspend the pellets, and the resulting mixture was incubated at 4 °C for 30 min. Afterwards, the solution was centrifuged at 1200 RPM for 5 minutes, from which the pellet was obtained and suspended in goat’s serum 3% (v/v) in human serum albumin/phosphate-buffered saline (PBS), followed by adding the antibody, including CD105 and CD34 phycoerythrin (PE-BL2+) conjugated antibodies, and CD73, CD90 and CD45 fluorescent-isothiocyanate (FITC-BL1+) conjugated antibodies, immediately, incubated on ice for 1 hour.

**Result:**

The analysis using flow cytometry was conducted. Afterwards, passage 3-isolated stem cells were tested for the expression of certain markers. Results from flow cytometry revealed that mesenchymal stem cell surface-specific markers like CD73 (100.0 %), CD90 (92.5 %), and CD105 (99.8 %) were expressed on bone marrow-derived stem cells while hematopoietic (non-mesenchymal) stem cell surface markers like CD34 (1.97 %) and CD45 (3.74 percent) were not.

***
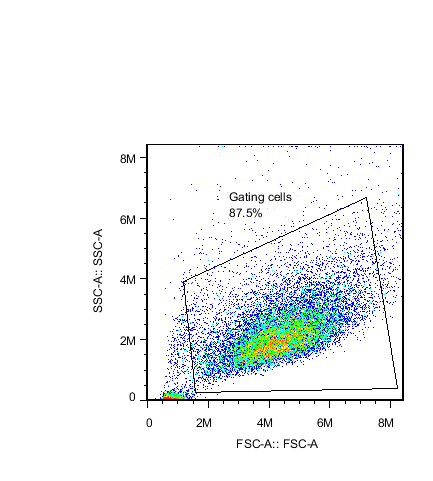
***

***(PLOT 1)***


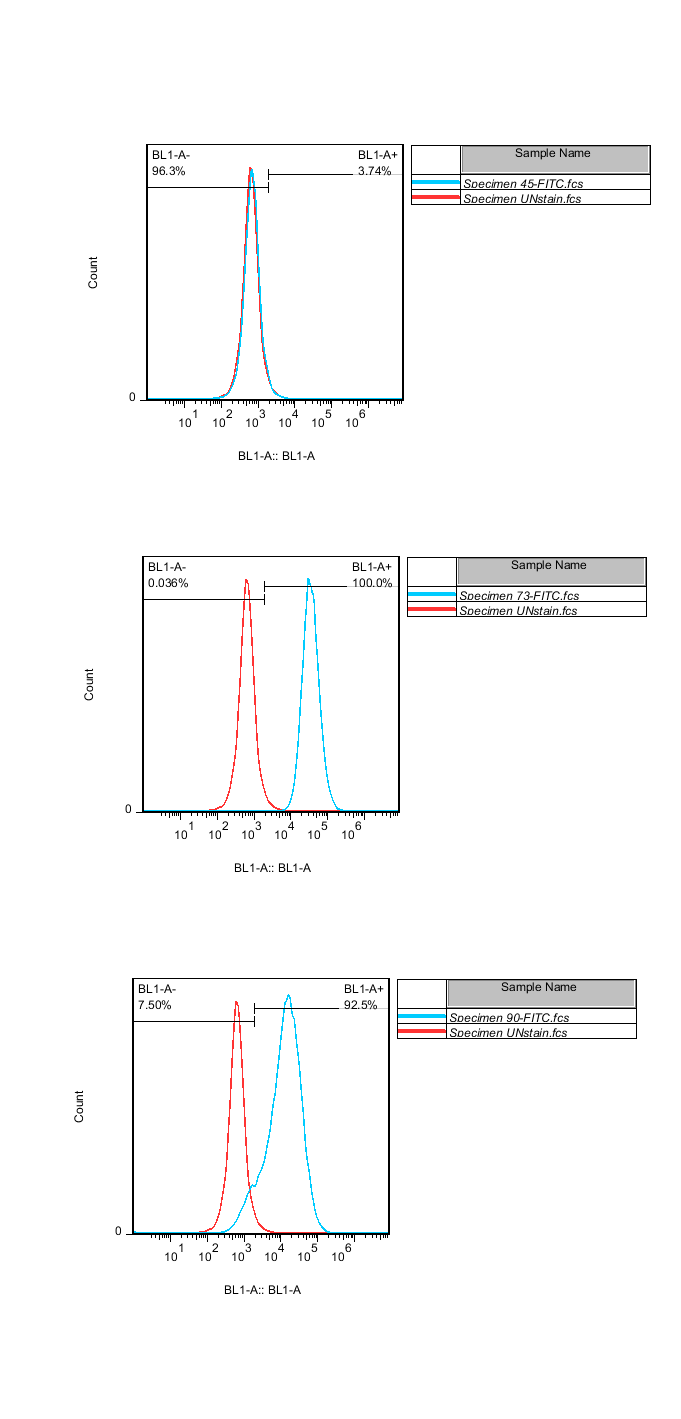

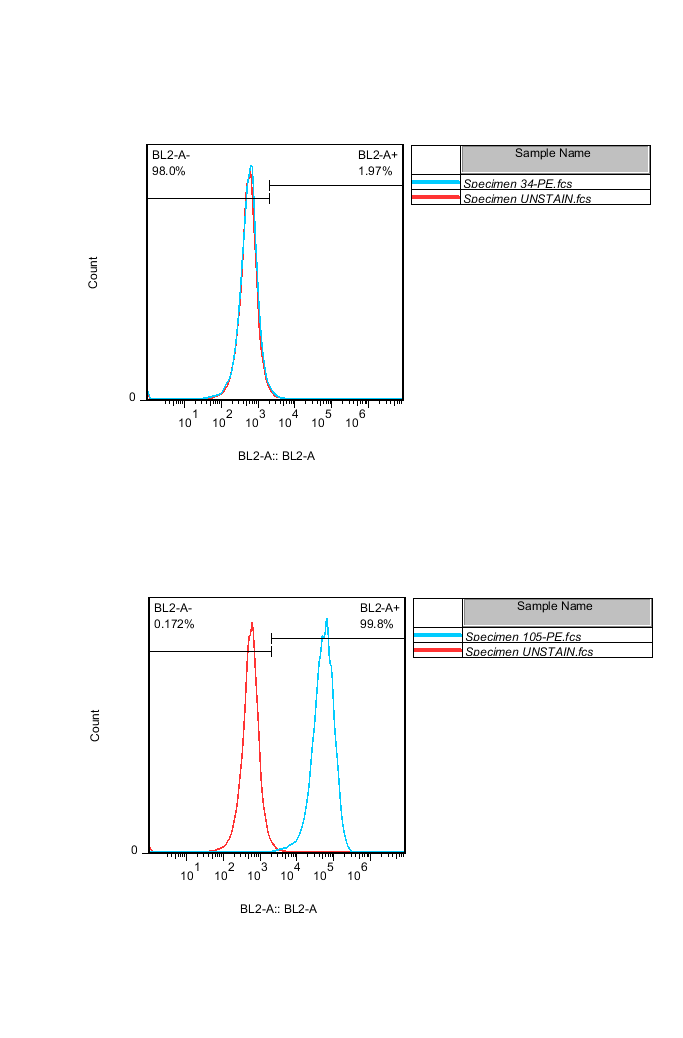


Figure 1. Figure 2.

1. Focal Adhesion Assay:

In order to quantify the Focal Adhesion, FA, of cells before and after plasma treatment use has been made of focal adhesion assay. The process may be broken down into the following steps:

The steps required to enable quantitative measurement of FA

Evaluating Focal adhesion quantitatively:

***** Utilize **SUBTRACT BACKGROUND** tool (Rolling Ball Background Subtraction is a plugin which tries to correct uneven illuminated background by using a “rolling ball” algorithm): Once the sliding paraboloid option is select, the rolling ball would become a parabolic with the same ball radius in pixels but a sharper curvature. A parabola slides across the image in multiple directions, calculating and subtracting local background from the main image. It will be ended up going with the *SLIDING PARABOLOID* option and a *ROLLING BALL* radius of 50 pixels.

***** Run CLAHE (Contrast Limited Adaptive Histogram Equalization) to improve the image's local contrast. To clarify desired objects, the plugin CLAHE provides 3 key parameters: block size, histogram bins, and maximum slope [1].

***** Use a mathematical exponential (EXP) to minimize the background even more.

***** Automatically adjust the BRIGHTNESS & CONTRAST. Based on an analysis of the image's histogram, the BRIGHTNESS & CONTRAST tool updates the lookup table.

***** Apply the LOG3D filter (Laplacian of Gaussian or Mexican Hat). The LOG3D plugin filters the image using user-defined parameters that are standard deviations in the X, Y, and Z directions. For 2D images, the Z direction is unimportant, and slice-by-slice processing is used [2].

***** Execute the THRESHOLD command. The THRESHOLD command converts a grayscale image to a binary image with 255 (white) and 0 pixel values (black). There are several threshold command options, including Huang, triangle, mean, and default. Furthermore, the user can automatically adjust the minimum and maximum threshold values or set the threshold levels based on an analysis of the histogram of the current image. In this case, we used the default method, and the threshold was automatically adjusted.

***** a- Using the "ANALYZE PARTICLES" command, b-scanning the thresholded (binary) image and finding the edges of objects or particles. Particle analyzers count and measure particles based on user-defined parameters such as size and circularity. The following parameters were established: size=50–infinity and circularity=0.00–0.99.

1. Saalfeld, S. "CLAHE (Contrast Limited Adaptive Histogram Equalization)." (2009).
2. Sage, Daniel, et al. "Automatic tracking of individual fluorescence particles: application to the study of chromosome dynamics." *IEEE transactions on image processing* 14.9 (2005): 1372-1383.
3. *Horzum, Utku, Berrin Ozdil, and Devrim Pesen-Okvur. "-by- quantitative analysis of focal adhesions." MethodsX 1 (2014): 56-59.*
